# The silent dialogue between cells: regulatory roles of non-coding RNAs in cardiovascular disease

**DOI:** 10.3389/fcell.2026.1921212

**Published:** 2026-09-21

**Authors:** Zhongwen Zhang, Yufei Yang, Mengzhen Liu, Hang Zhang, Zhaoyu Li, Jingjing Zhang, Haolong Zhang

**Affiliations:** 1 School of Public Health, Gansu University of Chinese Medicine, Lanzhou, Gansu Province, China; 2 Clinical College of Traditional Chinese Medicine, Gansu University of Chinese Medicine, Lanzhou, Gansu Province, China; 3 College of Acupuncture-Moxibustion and Tuina, Gansu University of Chinese Medicine, Lanzhou, Gansu Province, China; 4 Fuwai Yunnan Hospital, Chinese Academy of Medical Sciences, Affiliated Cardiovascular Hospital of Kunming Medical University, Kunming, China; 5 Yunnan Provincial Cardiovascular Clinical Medical Center, Kunming, China; 6 Yunnan Provincial Cardiovascular Clinical Medical Research Center, Kunming, China

**Keywords:** biomarkers, cardiovascular disease, individualized therapy, molecular regulation, non-coding RNA, precision medicine

## Abstract

Non-coding RNAs (ncRNAs) are involved in the occurrence, progression, and repair of cardiovascular disease By post-transcriptional regulation, epigenetic modification, cell-fate determination, and metabolic homeostasis-related pathways, microRNAs (miRNAs), long non-coding RNAs (lncRNAs) and circular RNAs (circRNAs) regulate the development of atherosclerosis heart failure and hypertension The stability in circulation and cell type–specific expression of these molecules have important implications for early disease detection, risk stratification, and prognostic prediction. The development of more sensitive detection techniques facilitates accurate quantification of ncRNAs in different sources including exosomes, plasma or tissues and promotes translational research. Interventions directed toward the regulation of ncRNAs, such as miRNA inhibition or replacement and modulation of lncRNAs and circRNAs are being developed. Despite existing challenges related to ncRNA delivery, specificity, and methodological standardization, ncRNAs represent promising molecular signatures for personalized medicine. Integrated use of multi-omics, clinical phenotypes and computational algorithms is anticipated to optimize an ncRNA-guided precision management pipeline from diagnosis to prediction and treatment.

## 1 Introduction

Studies on ncRNA have transformed our understanding of cardiovascular medicine. The development of high-throughput sequencing, single-cell omics, and spatial transcriptomics has revealed the regulatory roles of ncRNAs in intracellular and intercellular signaling (Yu et al., 2022; Liu M. et al., 2021). These discoveries highlight ncRNAs as critical contributors to disease progression. The pathophysiology of CVD is characterized by an intricate web of inflammation, metabolic stress, programmed apoptosis, fibrosis, and impaired tissue repair. NcRNA networks orchestrate these cellular and molecular functions (Liao et al., 2023). Emerging evidence shows that ncRNAs exist in complex networks across cells, tissues, and organs via exosome transportation. This intercellular exchange offers a molecular-level interpretation for disease heterogeneity (An et al., 2019).

Conventional clinical measures are required for risk stratification and monitoring of disease. Nevertheless, they are not adequate for monitoring dynamic, subtle pathophysiological changes and do not entirely account for individual heterogeneity. However, unlike traditional protein biomarkers, ncRNAs are characterized by cell-type specificity, temporal regulation, and resistance to degradation in body fluids. Expression patterns are altered early in the course of disease, even before morphologic injury occurs (Xue P. et al., 2023; zhai et al., 2021). These features confer utility to ncRNAs in early-stage diagnosis, stratification of diseases, and survival prediction. Therefore, the present review collects recent progress on miRNA, lncRNA, and circRNA in the field of cardiovascular medicine. This review addresses the regulation of these molecules, their diagnostic relevance, and therapeutic potential, as well as their application in personalized treatment approaches (Wu et al., 2018).

NcRNAs comprise a class of RNAs that are non-protein-coding but function as multilayered regulators. Among these, miRNAs are the most well-characterized subtype. They regulate post-transcriptional gene expression by interacting with specific messenger RNAs (mRNAs). As a class of RNA molecules, lncRNAs feature complex secondary structures and multifarious functions. They act as scaffolds, guides, or signal integration platforms to modulate chromatin architecture, transcriptional activity, and molecular interactions. CircRNAs are characterized by a covalently closed loop structure, which endows them with high stability. These molecules serve as miRNA sponges, interact with proteins, and modulate transcription (Fasolo et al., 2021; Yin et al., 2021).

These ncRNAs exhibit cell-type-specific expression profiles and participate in stress-responsive remodeling across major cardiac cell populations after myocardial injury (Maji et al., 2025). In addition, exosome-borne trafficking expands their regulatory range. This places ncRNAs as important molecular nodes in the coordination of local and systemic pathogenic processes (Yu H. et al., 2025; Wang R. et al., 2025; Liu J. et al., 2024).

CVDs remain a leading cause of global morbidity and mortality (Ye et al., 2023; Schlosser et al., 2020). Their pathogenesis is characterized by converging disruptions in energy metabolism, mitochondrial function, inflammation, cell death pathways, and fibrotic remodeling (LI et al., 2019; Liu et al., 2025; Guo et al., 2026). Even in patients with similar cardiovascular presentations, ncRNA profiles may reveal clinically relevant molecular heterogeneity, including differences associated with antiplatelet response and long-term prognosis (Rincón et al., 2022; Zhang Q. et al., 2022). Currently, no single biomarker is sufficient for the dynamic assessment of disease activity and interindividual heterogeneity, which remains a barrier to translational cardiovascular precision medicine. While genes and proteins are functionally rich, ncRNAs respond more rapidly to disease stimuli and offer distinct advantages for disease classification (Yao et al., 2018). Recent evidence suggests that ncRNAs act at important nodal points of signaling networks to modulate cellular responses to ischemia, inflammation, oxidative stress, mechanical load, and metabolic derangements (Liao et al., 2023; Zhang X. et al., 2022). MiRNAs are the best-characterized layer of regulation in the cardiovascular system. They regulate cardiomyocyte survival, calcium cycling, lipid metabolism, and vascular function, contributing to vasculopathy, AS, and myocardial infarction (MI) (Traxler et al., 2023; Wang JN. et al., 2016). LncRNAs are highly tissue- and context-specific. They are involved in the regulation of stress-responsive transcriptional programs, fibroblast activation, immune cell polarization, and vascular remodeling (Zhang M. et al., 2017; ZhanG and Bian, 2020; Li S. et al., 2021). The functions of circRNAs in ischemic repair, fibrotic signaling, and inflammation have also been elucidated; their structural integrity qualifies them as stable biomarkers and therapeutic candidates (Zhou Y. et al., 2024; Liu X. et al., 2021).

Different ncRNA classes crosstalk to form multilayered RNA-based regulatory networks. These encompass epigenetic, transcriptional, and post-transcriptional regulation (Zhang et al., 2026a; Zhang W. et al., 2025). They reflect cellular adaptation to environmental and biochemical cues (Zhai et al., 2020). As integrative markers of mechanistic understanding and translational utility, they can inform disease classification, risk prediction, and targeted intervention. With progress in multi-omics technologies and translational research, ncRNAs are evolving from mere diagnostic readouts into potential therapeutic targets. This transition offers new insights into biology and intervention for precision cardiovascular medicine (Bartman et al., 2017; Devaux et al., 2016).

## 2 Biological functions of ncRNAs

In the cardiovascular field, ncRNAs create a multilayered, complicated system that governs cell-type responses to heart-related stress conditions (Maji et al., 2025). These molecules represent a continually changing regulatory system, affecting transcriptional output, RNA stability, and RNA-protein interaction networks that enable disease progression (Aghagolzadeh et al., 2023; Pan et al., 2024). NcRNAs mediate complementary responses in cardiomyocytes, vascular endothelial cells, vascular smooth muscle cells (VSMCs), and immune cells to ischemic injury, vascular remodeling, and inflammation (Maji et al., 2025; Pan et al., 2024; Gong et al., 2021). Functionally, miRNAs provide rapid molecular feedback. LncRNAs act as scaffolds and functional modulators. CircRNAs feature a covalently closed structure, which ensures their stability under sustained cellular stress. By acting in concert, these classes of ncRNA establish a dynamic and integrated regulatory setting that is essential to the pathophysiology of the heart and vessels. Figure 1 presents the genomic source and structural/functional characteristics of ncRNAs, providing context for the intracellular regulatory networks these molecules underpin.

**FIGURE 1:**
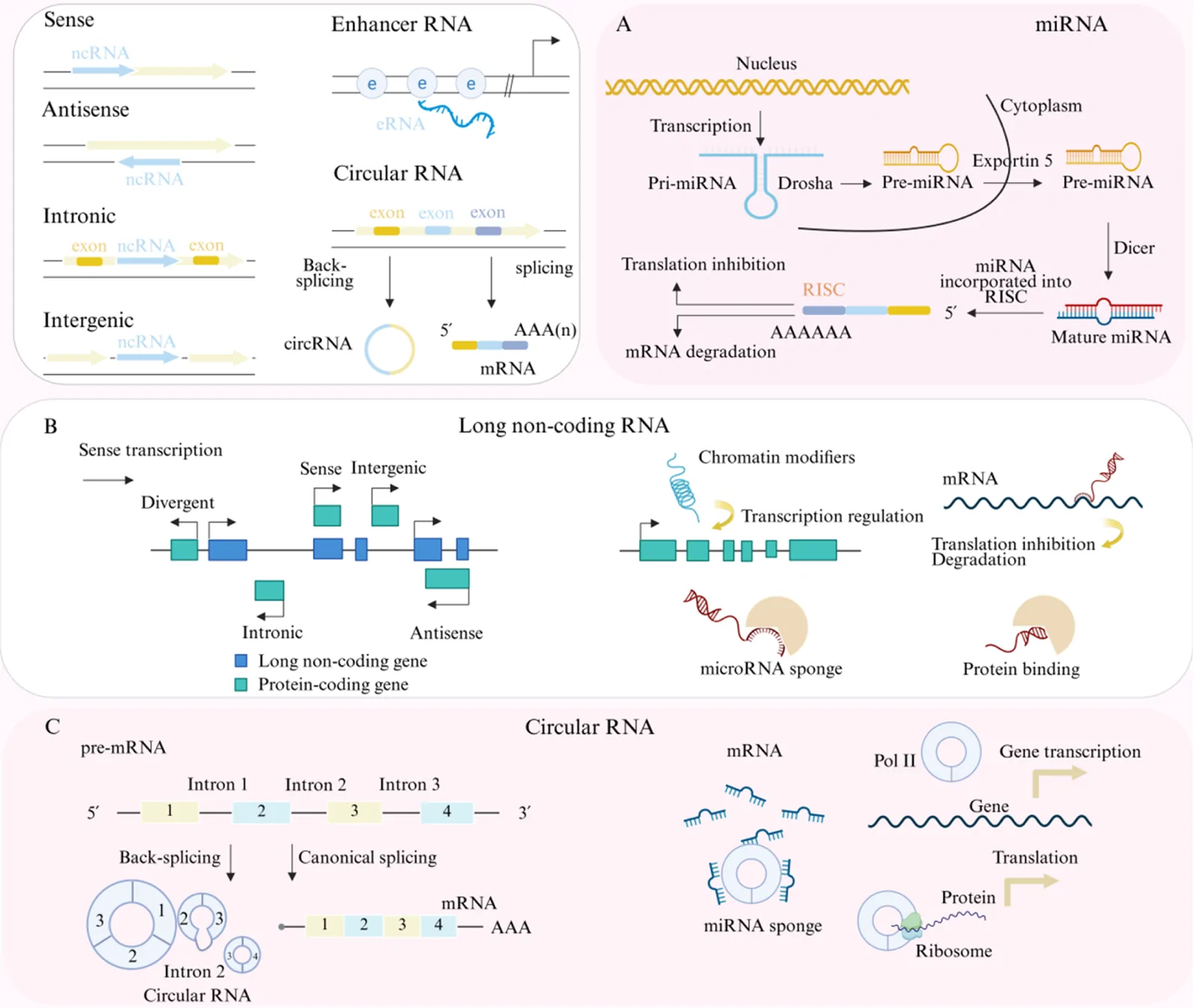
Classes of ncRNAs and their molecular features. This figure summarizes the genomic origins, biogenetic routes, and functional properties of major ncRNA species. The left panel illustrates the genomic contexts of ncRNA transcripts, including sense, antisense, intronic, intergenic, and enhancer-associated ncRNAs, as well as the formation of linear and circular RNA structures. **(A)** miRNA biogenesis involves nuclear processing of pri-miRNAs, export of pre-miRNAs to the cytoplasm, Dicer-mediated maturation, and incorporation of mature miRNAs into the RNA-induced silencing complex (RISC), leading to mRNA degradation or translational inhibition. **(B)** lncRNAs arise from diverse genomic contexts and regulate gene expression through chromatin modification, transcriptional regulation, modulation of mRNA stability and translation, miRNA sponging, and protein binding. **(C)** circRNAs are generated through back-splicing and can function as miRNA sponges, transcriptional regulators, or templates for translation. Collectively, these ncRNA classes form an interconnected regulatory network that modulates gene expression and cellular phenotypes.

RNA-binding proteins (RBPs) constitute another regulatory layer and assemble into ribonucleoprotein complexes and globally regulate RNA splicing, transport, stability, localization, and translation of coding and ncRNAs. A systematic census defined 1,542 human RBPs and indicated the widespread participation of RBPs in post-transcriptional gene regulation (Gerstberger et al., 2014). This concept has been further extended by new RNA interactome studies, which are also uncovering noncanonical RBPs lacking classical RNA-binding domains (Hentze et al., 2018). RBPs represent an alternative mechanism linking ncRNA regulation with pathological remodeling in CVD. Representative RBPs, including HuR/ELAVL1, IGF2BP family proteins, QKI, RBM20/RBM24, and hnRNPs, may influence hypertrophy, fibrosis, inflammation, ischemic injury, and HF by regulating alternative splicing, RNA stability, m6A-dependent RNA fate, and translational efficiency (Acharya et al., 2024). Hence, ncRNAs ought not to be thought of as single and linear regulators of target mRNAs in isolation but rather act as elements within dynamic ncRNA-RBP regulatory networks.

Evidence from cell and animal models should be interpreted as evidence for pathway regulation or candidate causal involvement, whereas findings primarily based on human samples should be interpreted as disease-associated expression patterns in the mechanistic tables that follow unless functional perturbation or disease-model validation is available. Thus, the listed ncRNAs should be considered graded mechanistic candidates or candidate therapeutic targets rather than uniformly validated therapeutic targets (Zhang W. et al., 2025; Mably and Wang, 2024).

### 2.1 Regulatory mechanisms and biological roles of miRNAs

Regarding ncRNA species, miRNAs operate as fast regulatory hubs allowing acute post-transcriptional regulation under cellular stress. They adjust protein translation and metabolic or survival pathways by binding to target mRNAs. For example, miR-129–5p regulates asporin (ASPN) and SOX9 to control fibrosis and cell reprogramming (Medzikovic et al., 2023), while small extracellular vesicles (sEVs) from fatty liver induce foam cell formation through the miR-30a-3p/ABCA1 axis (Chen X. et al., 2023). Similarly, miR-125 b targets multiple genes to suppress myocardial remodeling and apoptosis (Wang Z. et al., 2025). Under metabolic stress, miR-369–3p drives macrophage succinate metabolism through GPR91 to inhibit diabetic AS (Rawal et al., 2024); the miR-450–5p/ACSL4 axis inhibits ferroptosis during ischemic injury (Yu Q. et al., 2024); and miR-3158–3p targets Nur77 to stimulate post-infarction angiogenesis (Liao et al., 2023).

In addition to stress responses, many miRNAs are modulators of lipid metabolism and cardiovascular remodeling. MiR-30c inhibits lipid synthesis by down-regulating MTTP (Irani et al., 2016); miR-1180–3p modulates extracellular matrix (ECM) proteins through the ETS1 axis (Li C. et al., 2025); and miR-103–3p adjusts the balance between apoptosis and autophagy via Hlf, thereby promoting HF progression (Xue P. et al., 2023). Furthermore, the suppression of miR-195–3p reduces pathological ECM accumulation (Carvalho et al., 2023). Downregulation of miR-199a-3p and miR-15a-5p is correlated with the activation of NF-κB (González-López et al., 2023), while miR-1322 mediates ischemic cardioprotection through LRP8/PI3K/Akt signaling (Wei and Cui, 2023). These results indicate that miRNAs are key nodes connecting inflammatory, apoptotic, metabolic, and fibrotic signaling. Their expression patterns are altered early, often before tissue damage becomes detectable, suggesting their potential as early biomarkers and therapeutic targets, as summarized in Table 1.

**TABLE 1:** Role of miRNA in CVDs.

miRNA	Target gene/pathway	Roles in CVDs	Clinical significance	References
miR-144–3p	SLC7A11/ferroptosis pathway	Regulates ferroptosis and alleviates myocardial ischemia-reperfusion injury (MIRI)	Potential therapeutic target for improving cardiac function	(Ye et al., 2023)
miR-15a-5p	IKKα/IKKβ/p65/NF-κB pathway	Inhibits inflammation and ox-LDL uptake, attenuating AS	Diagnostic marker for advanced AS	(González-López et al., 2023)
miR-103–3p	Hlf/apoptosis–autophagy pathway	Promotes cardiomyocyte apoptosis and suppresses autophagy, aggravating HF	Potential therapeutic and diagnostic biomarker	(Xue et al., 2023a)
miR-33–5p	Fibrosis gene/Timp3–Mmp12 pathway	Regulates macrophage lipid metabolism and collagen production to improve plaque stability	Targeted inhibition may promote AS regression	(Zhang et al., 2022b)
miR-206	NFAT5/CCND2/SMAD2/Wnt pathway	Promotes AS progression and cardiovascular injury	Potential biomarker for predicting CVD risk after PE	(Schlosser et al., 2020)
miR-30a-5p	ABCC1/ABC transporter pathway	Regulates intestinal microbiota homeostasis and attenuates MI injury	Potential therapeutic target for maintaining gut–heart axis homeostasis	(Wang et al., 2025a)
miR-5099–3p	ELF1/pyroptosis pathway	Promotes cardiomyocyte pyroptosis in MI	Potential therapeutic target	(Yu et al., 2025a)
miR-24–3p	Top1/inflammatory pathway	Enhances inflammation and oxidative stress, increasing CVD risk	Potential target for CVD intervention	(Zhang et al., 2025a)
miR-143/145 cluster	Regulates phenotypic switching of VSMCs	Involved in AS; protective against CAD	rs41291957 is a CAD-protective factor	(Hall et al., 2021)
miR-23a, miR-92a	VEGF regulation/anti-apoptotic pathway	Inhibits cardiomyocyte apoptosis after MI	Facilitates BM-MSC–mediated cardiac recovery after MI	(Song et al., 2017)
miR-221/222 family	Targets TGF-β–related genes (e.g., JNK1)	Downregulates myocardial fibrosis and functional deterioration	Associated with HF, negatively correlated with fibrosis severity	(Verjans et al., 2018)
miR-616–3p	RFX7 pathway	Mediates ox-LDL–induced HUVEC injury; involved in AS	Key regulatory factor associated with AS	(Yu et al., 2022)
miR-27a	CSE/H2S signaling pathway	Upregulation promotes cardiac hypertrophy; inhibition is protective	Potential therapeutic target for cardiac hypertrophy	(Gao et al., 2024)
miR-323a-3p	TIMP3/TGF-β pathway	Upregulation promotes myocardial fibrosis; inhibition improves fibrosis	Biomarker and therapeutic target for myocardial fibrosis	(Zhang et al., 2018a)
miR-126, miR-223	COX2	Downregulated in CA; associated with plaque stability	Diagnostic indicators for CA and plaque stability	(Zhu et al., 2023c)
miR-99b-3p	GSK-3β/Smad3 pathway	Upregulated by Ang II; promotes myocardial fibrosis	Potential target for fibrosis-related cardiomyopathy	(Yu et al., 2021)
miR-130b-3p	ACSL4/PRKAA1/AMPK–mTOR pathway	Improves sepsis-induced cardiomyopathy by inhibiting ferroptosis	Potential therapeutic target for septic cardiomyopathy	(Qi et al., 2023)
miR-146a	IRAK1/TRAF6/NF-κB pathway	Reduces inflammation and apoptosis in MIRI; improves cardiac function	Potential targeted-delivery strategy for MIRI therapy	(Meng et al., 2024)
miR-425–5p	TGF-β1/Smad pathway	Downregulated in HF; overexpression attenuates fibrosis and HF	Diagnostic and therapeutic biomarker for HF	(Zhou et al., 2024b)
miR-574–3p	IL-6/IL-6–JAK–STAT pathway	Upregulated during exercise; protects against pressure overload–induced hypertrophy	Mediates exercise-induced cardioprotection; valuable for cardiac rehabilitation	(Chen et al., 2024b)
miR-133a	Ca2+/calcineurin/NFATc4 pathway	Downregulated in hypertrophy; upregulation suppresses hypertrophy	Mediates H2S anti-hypertrophic effects	(Wu et al., 2018)
miR-148a-3p	PCSK9; NF-κB inhibition	Protects endothelial cells and alleviates CAD	Provides new therapeutic insights for CAD	(Tang et al., 2024)
miR-124	Akt1/PI3K–Akt pathway	Upregulated in VD; inhibition improves cognitive function	Potential therapeutic target for VD	(Liu et al., 2024b)
miR-181a-5p	CD93/SLC7A11 signaling	Promotes endothelial ferroptosis/inflammation, accelerating AS	Potential target for AS treatment	(Gu et al., 2024)
miR-17–5p	PCSK9/VLDLR pathway	Downregulation suppresses inflammation and slows AS progression	Potential target for AS prevention/treatment	(Qin et al., 2022)
miR-21	RECK, PDCD4, TGF-β pathway	Promotes myocardial fibrosis	Plasma biomarker for myocardial fibrosis	(Villar et al., 2013)
miR-15a, miR-146a	VEGF/PI3K/eNOS axis	Upregulated after MI; inhibition improves function and angiogenesis	Supports ART + VD3 therapy post-MI	(Saati-Zarei et al., 2023)
miR-19a-3p	PIK3CA/PI3K–AKT pathway	Inhibition promotes angiogenesis and improves MI/R injury	Provides new mechanism for MI/R protection	(Liu M. et al., 2021)
miR-125a-5p	TRAF6/TRAF6–IRF5 pathway	Promotes macrophage M2 polarization and AMI repair	Potential translational target for AMI	(Gong et al., 2024)
miR-3574	Axin1	Downregulated in IH; overexpression alleviates injury	Therapeutic target for obstructive sleep apnea (OSA)-related CVD	(Chen et al., 2021c)

Furthermore, miRNAs serve as master regulators of cardiovascular homeostasis that help to integrate signaling in metabolic, inflammatory, and cell death pathways. Nevertheless, there are considerable differences in how specific miRNAs impact biology across different studies. For example, miR-125 b shows context-dependent roles in cardiomyocyte injury, fibrosis, and cardiac metabolism (Chen C-Y. et al., 2021), while miR-30a-3p and miR-199a-3p also present opposing regulatory effects depending on tissue type. This characteristic reflects their crosstalk with different cellular networks. The multi-target nature of miRNAs extends their regulatory potential; however, it also challenges mechanistic interpretation and raises safety and translational concerns (Huang et al., 2025a). Present evidence remains predominantly correlative and lacks causal validation in a quantitative or dose-response fashion. Therefore, the clinical application of miRNAs must be based on systems-level integration and high-quality experimental verification.

### 2.2 Roles of lncRNAs in cardiovascular development and pathology

In the case of lncRNAs, they also display striking tissue specificities and structural complexities; their function is now known to be much more than that of transcriptional by-products. LncRNAs govern transcription, post-transcriptional processing, and signaling dynamics via interactions with DNA, RNA, and protein complexes. For example, Malat1 regulates mitochondrial fission through the miR-26b-5p/Mfn1 axis (Chen Y. et al., 2021); MIR181A1HG targets Foxp1 to enhance NLRP3 inflammasome activity, leading to vascular inflammation (Ni et al., 2025); GAS5 modulates endothelial cell viability via miR-194–3p (Li et al., 2021b); and Swhtr interacts with NKX2-5 to regulate hypoxia sensitivity (Rogala et al., 2023).

In atherosclerotic and inflammatory systems, lncRNAs are system-level regulators. MALAT1 is involved in foam-cell formation by modulating the miR-330–5p/NF-κB axis (Shi et al., 2021). H19 is downregulated under ischemic conditions; when overexpressed, it has been shown to promote autophagy and improve cardiac function (Zhou et al., 2018), although other findings suggest it promotes fibroblast proliferation and fibrosis (Tao et al., 2016). SNHG15 directly modulates cardiomyocyte apoptosis in response to ischemia-reperfusion injury (IRI) (Chen D. et al., 2023), Oip5-as1 regulates mitochondrial fission via the AKAP1/CaN axis (Niu et al., 2024), and suppression of CCAT2 attenuates myocardial hypertrophy by blocking Wnt/β-catenin signaling (Zhang et al., 2024). Additionally, KCNQ1OT1 exacerbates cardiac dysfunction through activation of the miR-204–5p/LGALS3 network (Rong et al., 2020), and MIAT promotes maladaptive remodeling by inhibiting miR-150 (Zhu et al., 2016). These lncRNAs modulate cellular stress responses through disease-dependent expression patterns and long-range regulation. Their stability and distinct regulatory functions make them central molecules in the modulation of fibrosis, inflammation, and tissue remodeling, as summarized in Table 2.

**TABLE 2:** LncRNA contributions to CVD pathogenesis.

lncRNA	Target gene/pathway	Roles in CVDs	Clinical significance	References
MIR181A1HG	Foxp1, NLRP3 inflammasome, NF-κB/p65	Promotes vascular inflammation and AS	Potential therapeutic target for vascular inflammatory diseases	(Ni et al., 2025)
MALAT1	UPF1, SIRT6, Wnt/β-catenin pathway	Promotes the development of viral myocarditis (VMC)	Potential therapeutic target for VMC	(Zeng et al., 2024)
MIAT	EGR1–ELK1–ERK pathway, KLF4	Upregulation activates abnormal SMC behavior, accelerating AS	Potential therapeutic target for AS	(Fasolo et al., 2021)
MAAMT	Interacts with SRSF1; regulates NF-κB pathway	Upregulation promotes macrophage pro-inflammatory activation and exacerbates cardiac injury	Provides new insights for early diabetic cardiomyopathy (DCM) therapy	(Gan et al., 2024)
H19	p38, p65 (protein expression)	Promotes HUVEC proliferation and inhibits apoptosis	Potential therapeutic target for AS	(Pan, 2017)
Gm2691	Akt signaling (linked with ERK1/2)	Improves cardiac function; reduces inflammation and apoptosis	Provides new direction for acute MI therapy	(Li et al., 2019)
ANRIL	Sponges miR-399–5p; regulates RAS/RAF/ERK pathway	Promotes AS-related cell proliferation and migration	Potential therapeutic target for AS	(Huang et al., 2020c)
lncARF	Binds RRAGD; activates PI3K/Akt and MAPK pathways	Promotes macrophage autophagy in foam cells and reduces atherosclerotic lesions	Biomarker for HHcy-related AS	(Ding et al., 2025)
PVT1	Sponges miR-30a; regulates Beclin-1 axis	Promotes ventricular remodeling and fibrosis in hypertensive rats	Target for HTN-related ventricular remodeling	(Ge et al., 2025)
AK020546	Sponges miR-350–3p; activates ErbB3	Attenuates myocardial I/R and oxidative stress injury	Potential therapeutic target for ischemic heart disease	(Zhang et al., 2021b)
SNHG16	Sponges miR-17–5p; activates NF-κB pathway	Promotes AS-related cell proliferation and inflammation	Potential therapeutic target for AS	(An et al., 2019)
NR_045363	Inhibits p53 pathway	Reduces cardiomyocyte apoptosis and improves cardiac function after MI	Potential target for myocardial repair	(Chen et al., 2020b)
TINCR	Sponges miR-211–3p; activates VEGFB–SDF-1α–CXCR4 axis	Suppresses myocardial hypertrophy progression	Potential therapeutic target for cardiac hypertrophy	(Tu et al., 2022)
AK087124	miR-224–5p/PTEN/AKT pathway	Promotes ox-LDL-induced endothelial apoptosis and inflammation	Highly expressed in plasma/plaques; diagnostic and therapeutic value	(Zhai et al., 2021)
CASC7	miR-21/PI3K–AKT; miR-21/TLR4–NF-κB pathways	Inhibits VSMC proliferation; promotes apoptosis; reduces HUVEC inflammation	Low serum expression; diagnostic and therapeutic potential	(Pei et al., 2021)
NEAT1	miR-214–3p/PTEN/PI3K–AKT–mTOR pathway	Promotes VIC osteogenic differentiation; involved in calcific aortic valve disease (CAVD)	Upregulated in calcified valves; diagnostic and therapeutic target	(Xu et al., 2025b)
PCFL	miR-378/GRB2	Promotes post-MI fibrosis and CF proliferation	Upregulated in MI; anti-fibrotic therapeutic target	(Sun et al., 2019)
ENST00113 (lnc00113)	PI3K/Akt/mTOR signaling	Promotes VSMC/HUVEC proliferation, survival and migration	Highly expressed in patient serum; therapeutic target for AS	(Yao et al., 2018)
GAS5	miR-21/PDCD4/PI3K–AKT pathway	Mediates MI-induced cardiomyocyte apoptosis	Downregulated in MI; potential therapeutic target	(Zhou et al., 2020a)
MALAT1	Notch signaling (Notch-1)	Reduces HTN-induced vascular remodeling and EC apoptosis	Upregulated in HTN; diagnostic and therapeutic marker	(Xue et al., 2019)
GASL1	PI3K/Akt pathway	Regulates HF progression; suppresses cardiomyocyte apoptosis	Downregulated in HF; therapeutic regulatory target	(Zhou et al., 2022b)
ZFAS1	miR-150–5p/Notch3 axis	Promotes ox-LDL-induced EndMT in HUVECs	Upregulated in AS; therapeutic target	(Yin et al., 2021)
GAS5	PTEN/MMP-2 pathway	Reduces ISO-induced cardiac fibrosis; improves cardiac function	Downregulated in fibrotic myocardium	(Liu et al., 2019)
SOX2-OT	miR-146a-5p	Reduces apoptosis, inflammation, oxidative stress in MIRI	Upregulated in MIRI; therapeutic target	(Li et al., 2023c)
ROR	miR-138/Mst1	Exacerbates hypoxia/reoxygenation (H/R)-induced cardiomyocyte injury	Therapeutic target	(Hu et al., 2020)
FOXD3-AS1	miR-128/TXNIP/Redd1–AKT–GSK3β–Nrf2 axis	Aggravates myocardial I/R and H/R injury; suppresses protective pathways	Target for myocardial protection	(Chen et al., 2022b)
RMRP	miR-128-1-5p/Gadd45 g pathway	Promotes coronary AS inflammation and VSMC apoptosis	Upregulated in coronary AS; therapeutic target	(An et al., 2020)
TUG1	miR-34a/DKK1/Wnt–β-catenin pathway	Reduces TAC/PE-induced hypertrophy	Upregulated in hypertrophy; therapeutic target	(Fang et al., 2020)
NORAD	miR-22–3p/PTEN/AKT–mTOR pathway	Promotes AMI progression; aggravates H/R injury	Highly expressed in AMI; therapeutic target	(Li et al., 2022b)
TTTY15	miR-374a-5p/FOXO1/autophagy	Exacerbates myocardial I/R injury via autophagy suppression	Therapeutic target	(Chen et al., 2021d)

Although the role of lncRNAs is recognized in cardiovascular regulation, these RNAs exhibit marked disease- and context-specific variation with respect to their biological functions (Cao et al., 2022). Molecules such as Malat1, GAS5, and Oip5-as1 have protective roles in metabolic homeostasis and cell survival, whereas H19, KCNQ1OT1, and MIAT mediate inflammation or structural remodeling under certain pathological contexts. This dichotomy emphasizes their context-dependent nature. Discrepancies between experimental models and an incomplete mechanistic understanding contribute to many causal pathways remaining unclear. The inherent complexity of lncRNAs provides an extended regulatory capacity but also complicates functional dissection and clinical application. It is necessary to characterize their precise functions in disease networks to determine whether these lncRNAs have clinical potential as biomarkers or therapeutic targets (Cao et al., 2022). Figure 2 illustrates the multi-scale regulatory networks built by ncRNAs in cardiovascular cells and their roles in homeostasis and pathological remodeling.

**FIGURE 2:**
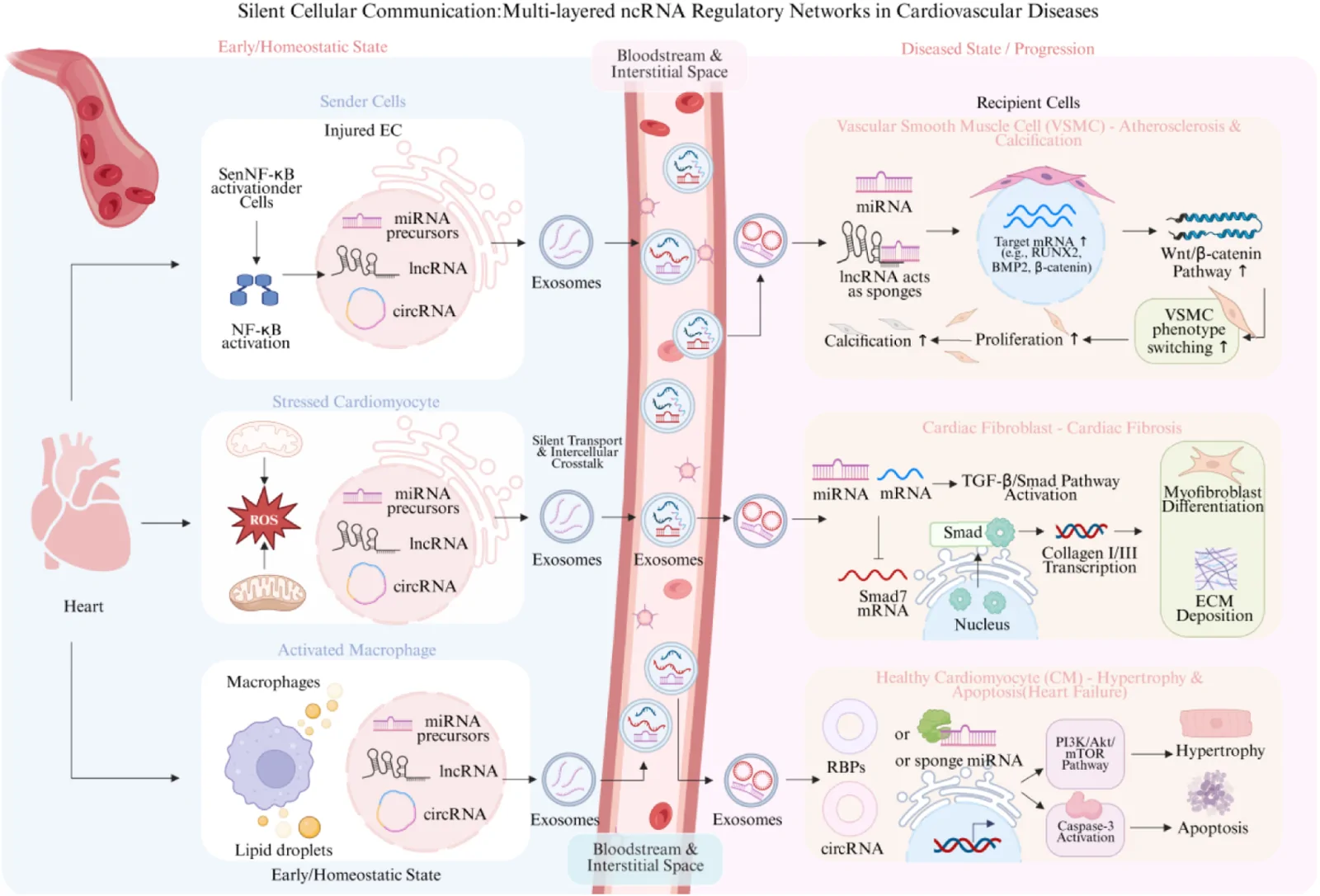
Hierarchical ncRNA regulatory networks across cardiovascular cell types. This figure shows the cross-cellular regulatory network formed by ncRNAs during the transition from cardiovascular homeostasis to disease. Injured endothelial cells, stressed cardiomyocytes, and activated macrophages release EVs enriched in miRNA precursors, lncRNAs, and circRNAs into the circulation and interstitial microenvironment. As these vesicles are taken up by target cells (VSMCs, fibroblasts, and healthy cardiomyocytes), ncRNAs reprogram recipient cell signaling in a sequence-specific manner. In smooth muscle cells, ncRNAs modulate the Wnt/β-catenin axis and calcification-related pathways, driving phenotypic switching. In fibroblasts, exogenous miRNAs modulate TGF-β/Smad signaling to promote collagen deposition and matrix remodeling. In cardiomyocytes, circRNAs cooperate with RBPs to regulate the PI3K/Akt/mTOR pathway and apoptotic programs. Collectively, these interactions form an ncRNA-mediated communication network that coordinates cardiovascular pathology across spatial and temporal scales, conferring both structure and plasticity to disease progression.

### 2.3 Distinctive properties and functions of circRNAs

The covalently closed loop structure of circRNAs confers resistance to exonucleolytic decay and supports sustained regulatory effects in cardiovascular contexts (Gong et al., 2021). Besides serving as miRNA sponges, circRNAs can regulate signaling through RNA-protein interactions and, in some cases, translation into functional micropeptides (Gong et al., 2021; Xu et al., 2022). For instance, circSCMH1 regulates metabolic homeostasis by affecting the nuclear translocation of FTO and m6A modification of Plpp3 (Li B. et al., 2023). Overexpression of circDICAR represses pyroptosis in diabetic conditions (Yuan et al., 2023); circEsyt2 regulates p53β alternative splicing in VSMC proliferation (Gong et al., 2021); and nanoparticle-mediated delivery of circZFPM2 attenuates hypertrophy while promoting cardiomyocyte survival (Neufeldt et al., 2024). In addition, FEACR modulates ferroptosis by inhibiting the NAMPT-Sirt1-FOXO1-FTH1 axis to attenuate IRI (Ju et al., 2023); circ-Ddx60 regulates eEF2 and AMPK activity (Zhu Y. et al., 2023); exosomal circWhsc1 enhances STAT3 activation for facilitating cardiac regeneration (Wei et al., 2023); while circNlgn encodes the micropeptide Nlgn173 to regulate inflammation and cell death through γH2AX signaling (Xu et al., 2022).

Increasing attention is focused on the correlation of circRNAs with myocardial ischemia, fibrosis, and tissue remodeling. CircCHSY1 maintains mitochondrial homeostasis dependent on HO1 (Tan et al., 2024); circITCH sponges miR-330–5p to increase SIRT6 and SERCA2a, thus attenuating doxorubicin (DOX)-induced damage (Han et al., 2020); circCacna1c inhibits RIPK1-mediated necroptosis and improves cardiac function (Jia et al., 2024); statin-induction of circRNA-RBCK1 enhances diastolic function in heart failure with preserved ejection fraction (HFpEF) by sponging miR-133a (LI B. et al., 2024); circRBAC regulates ribosome biosynthesis mediated by Ddx21 (Huang et al., 2025b); and circARCN1 accelerates the atherogenic process through the HuR/USP31/NF-κB axis (Pan et al., 2024). Their molecular stability, cell-type selectivity, and ability to be transported by exosomes identify circRNAs as stable regulators and translatable disease markers. This changes the view of how phenotype modulation and system-level signaling are executed in CVDs (Table 3).

**TABLE 3:** CircRNA-related mechanisms in cardiovascular pathologies.

circRNA	Target gene/pathway	Disease mechanism	Clinical significance	References
circNfix	Ybx1/Nedd4l; miR-214/Gsk3β/β-catenin	Inhibits cardiomyocyte proliferation, promotes apoptosis, suppresses angiogenesis after MI	Downregulation improves post-MI cardiac function	(Huang et al., 2019)
circNSD1	miR-429–3p/SULF1/Wnt–β-catenin	Promotes myocardial fibrosis after MI	Therapeutic target for cardiac fibrosis	(Ji et al., 2024)
circ_0008362	miR-1251–5p/Runx2	Promotes VSMC calcification in diabetic arterial calcification	Potential therapeutic target	(Lin et al., 2024)
circDGKZ	miR-345–5p/TLR4/NF-κB/NLRP3	Promotes cardiomyocyte pyroptosis; inhibits autophagy in MIRI	Potential therapeutic target	(Li et al., 2024b)
circCHSY1	miR-24–3p/HO1	Cardioprotective during I/R by maintaining mitochondrial homeostasis	Potential protection target for I/R injury	(Tan et al., 2024)
circ-Stt3b	miR-15a-5p/GPX4	Reduces apoptosis, ROS and ferroptosis after MI	Helps repair myocardial injury post-MI	(Liu et al., 2024a)
circRNA-0044073	miR-107/JAK–STAT	Promotes proliferation, invasion, inflammation in AS	Novel therapeutic target	(Shen et al., 2019)
circ_0079480	miR-338–3p/THBS1/TGF-β1/Smad3	Promotes atrial fibrosis in atrial fibrillation (AF) (Ang II-induced)	Target for AF-related fibrosis therapy	(Wei et al., 2024)
circDhx32	FOXO1/AdipoR1–AMPK–NF-κB	Promotes inflammation in myocardial I/R	Therapeutic target for ischemic heart disease	(Si et al., 2025)
circRNA_000203	miR-26b-5p/miR-140–3p/Gata4	Promotes cardiac hypertrophy	Therapeutic target for hypertrophy	(Li et al., 2020a)
circRNA CHRC	miR-431–5p/Klf15	Suppresses hypertrophy by maintaining Klf15	Therapeutic target	(Hu et al., 2024b)
circ_SMG6	miR-138–5p/EGR1/TLR4/TRIF axis	Promotes neutrophil recruitment in I/R injury	Target for myocardial I/R therapy	(Huang et al., 2022b)
circJARID2	miR-9-5p/BNIP3 axis	Upregulated in hypoxia; promotes apoptosis and inflammation	Inhibition reduces ischemic injury	(Cai et al., 2021)
circFASTKD1	miR-106a/LATS1/2/YAP	Suppresses angiogenesis in ECs	Silencing improves cardiac function post-MI	(Gao et al., 2020)
circ_0062389	TGF-β1/Smad3	Reduces cardiomyocyte apoptosis	HF therapeutic target	(Zhang and Chen, 2021)
circHDAC9	miR-671–5p/SOX4	Promotes myocardial I/R injury	Therapeutic target	(Liu et al., 2024c)
circRNA-0006896	miR-1264–DNMT1/JNK–STAT3	Regulates endothelial dysfunction and plaque instability	AS therapeutic target	(Wen et al., 2021)
circRbms1	miR-92a/BCL2L11	Acute MI	Therapeutic strategy target	(Jin et al., 2022)
circ_Malat-1	NF-κB signaling	Inhibited by GDF15; prevents transplant rejection	Protective factor for cardiac transplantation	(Zhang et al., 2018b)
circ_0073932	miR-493–3p/FAF1/JNK	Mediates apoptosis and oxidative stress	New therapeutic target for I/R	(Su et al., 2024)
circRTN4	miR-497–5p/MG53	Sepsis-induced cardiac injury; alleviates apoptosis, oxidative stress, inflammation	Therapeutic potential for septic cardiomyopathy	(Li et al., 2022c)
circ_0060745	NF-κB pathway	Inhibits apoptosis and inflammation	AMI therapeutic target	(Zhai et al., 2020)
circANRIL	PES1/rRNA maturation/p53	AS	Protective regulator in AS	(Holdt et al., 2016)
circ-Ttc3	miR-15b-5p/Arl2	Anti-apoptotic cardioprotection	Protective target for MI	(Cai et al., 2019)
hsa_circ_0007623	miR-297/VEGFA	Promotes myocardial repair	Potential target for ischemic heart therapy	(Zhang et al., 2020)
circNfix	miR-145–5p/ATF3	Reduces hypertrophy	Hypertrophy therapeutic target	(Pan et al., 2021)
circ-HIPK2	miR-485–5p/ATG101	Promotes autophagy and apoptosis	Therapeutic target for myocardial injury	(Zhou et al., 2020b)
circHIPK3	miR-29a/VEGFA	Promotes angiogenesis	Target for angiogenesis after MI	(Wang et al., 2020b)
circ_GRN	miR-214–3p/FOXO1	Promotes VSMC proliferation, migration, inflammation in AS	New target for AS intervention	(Li et al., 2021g)
circ_0001148	miR-218–5p/JMY	Promotes EndMT and plaque formation in AS	Therapeutic target for AS	(Yu et al., 2025b)

Despite the necessity for more experimental evidence to support the functional roles of circRNAs, they are considered stable members of cardiovascular signaling molecules. The protective function of some circRNAs in ischemia or remodeling may coexist with inflammatory and pathological promoting roles, reflecting a context-dependent nature. Studies have mainly focused on individual molecular interactions, especially miRNA-sponging mechanisms, whereas circRNA biogenesis, transcriptional regulation, and cooperative networks remain incompletely defined (Cheng et al., 2024). Although stability is generally an asset, it can generate ambiguity when attempting to interpret changes in regulation under dynamic conditions. A critical question is whether circRNAs serve as causal mechanistic nodes rather than associative signal markers. This distinction is critical to determine their applicability to precision cardiovascular medicine. The comprehensive miRNA-lncRNA-circRNA interaction network and its synergistic regulation of key cardiovascular target genes are presented in Figure 3.

**FIGURE 3:**
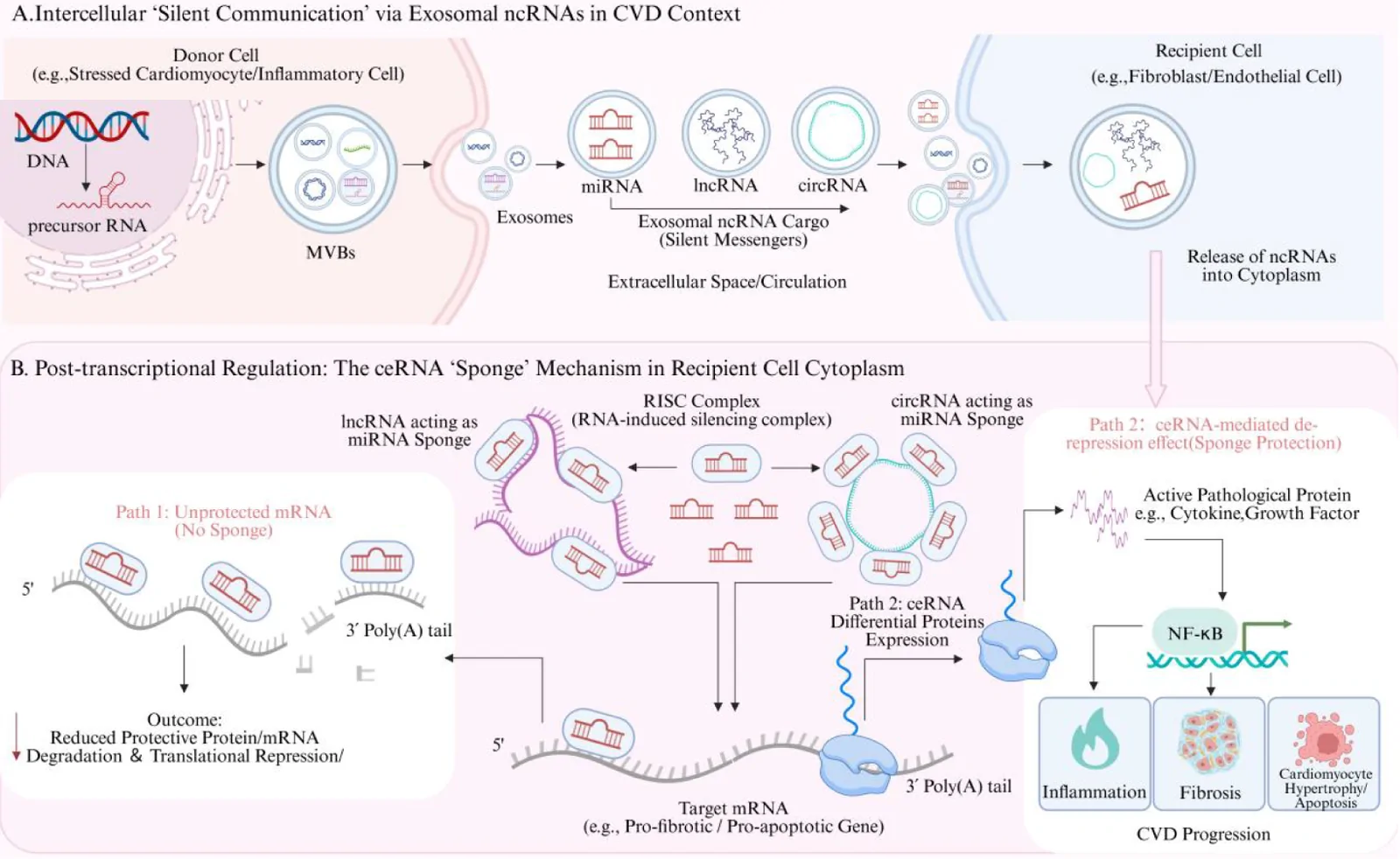
Interaction networks and targeting architectures among miRNAs, lncRNAs, and circRNAs. This figure illustrates the intercellular transfer and intracellular regulatory interactions of ncRNAs in the cardiovascular disease (CVD) context. **(A)** Stressed cardiomyocytes or inflammatory cells package miRNAs, lncRNAs, and circRNAs into exosomes, which are released into the extracellular space or circulation and subsequently taken up by recipient cells, such as fibroblasts and endothelial cells. Exosomal ncRNAs are then released into the cytoplasm, enabling intercellular communication between donor and recipient cells. **(B)** In recipient cells, lncRNAs and circRNAs can act as competing endogenous RNAs (ceRNAs) by binding shared miRNAs and thereby modulating miRNA-mediated post-transcriptional repression. In the absence of effective miRNA sponging, miRNA-loaded RISC promotes target mRNA degradation or translational repression. In contrast, ceRNA-mediated sequestration of miRNAs can relieve repression of target mRNAs, increase the expression of pathological proteins, and activate downstream signaling pathways such as NF-κB, thereby contributing to inflammation, fibrosis, cardiomyocyte hypertrophy, apoptosis, and CVD progression.

## 3 Biomarker potential of ncRNAs

In studies on CVDs, ncRNAs can be found with tissue-specific, dynamic, and measurable expression patterns. These profiles pertain to major pathological processes, such as cell damage, inflammation, fibrosis and tissue healing. Exosomal encapsulation, protein binding, as well as their structural stability are however the cause of persistence of ncRNAs in circulation making them useful molecular candidates for early detection, risk stratification and monitoring treatment. Therefore, this chapter systematically reviews early diagnosis, detection technologies and translational research opportunities.

### 3.1 Early diagnosis and prognostic assessment

The levels of ncRNAs in AS, HF, MI, and inflammatory CVD are related to the severity of the disease. The structural changes reported here can occur early, before the onset of direct structural damage, thus overcoming the limitation of classic biomarkers. In AS, lncSOX2-OT is highly induced in patients with carotid plaques correlating well with lesion progression and suggesting useful potential as a marker of disease severity (Tao and Hu, 2022). LncRMRP resists the p53 stress signaling and strengthens the insult response to induce cardioprotection further confirming its mechanistic potential as a biomarker (Li J. et al., 2023). Moreover, the expression level of THBS1-AS1 is positively associated with the degree of cardiac fibrosis, indicating its potential as a therapeutic target for tracking fibrotic progression and finding the best therapeutic windows (Zhou et al., 2023).

MiRNAs provide several benefits for the early diagnosis of acute cardiovascular events. High levels of miR-146a in ST-segment-elevation myocardial infarction (STEMI) patients are associated with major adverse cardiovascular events (MACE), and mechanistically, this molecule could modulate disease progression by a S100A12-induced inflammatory pathway (Xiao et al., 2021). Refinements in established risk models are achieved through multimarker miRNA panels which further predict HF hospitalization, cardiovascular mortality and New York Heart Association (NYHA) functional class (Rincón et al., 2022). In hemodialysis patients, circulating miR-122–5p reflects hepatic activity and cardiac injury, thereby helping guide clinical management in critically ill patients (Duni et al., 2023).

In immune-inflammatory signaling, the atherosclerotic role of lncARF is through the RRAGD/PI3K/Akt and RRAGD/MAPK pathways (Ding et al., 2025). In contrast, miR-155 is downregulated in the process of plaque regression but enriched in urinary exosomes of unstable coronary artery disease (CAD) patients; thus, it may be useful for identifying plaque destabilization and predicting prognosis (Fitzsimons et al., 2020). Relationship between MALAT1 expression and infarct size as well as inflammatory burden in patients with acute myocardial infarction (AMI) has been established but remains to be validated in larger cohorts (Li R. et al., 2022). In ischemia and cardiac repair, knocked down expression of circNfix blocks the degradation of Ybx1 that leads to enhancing bifunctional miR-214 activity, which ultimately promotes post infarction regeneration and functional recovery (Huang et al., 2019). Also, the circIGF1R/DXX5/β-catenin axis contributes to ischaemia repair through augmented cardiomyocyte regeneration and structural recovery (Shan et al., 2024).

In MI, HF and AS, temporal changes in ncRNA expression are associated with disease progression, and these ncRNAs may serve as sensitive, non-invasive biomarkers with diagnostic and prognostic value (Caporali et al., 2024). Table 4 displays the studies described in this section, including molecular classes, detection approaches, corresponding disease states, clinical utilities, and grades of evidence. Accordingly, pathway regulation or candidate causal roles are primarily supported by cell and animal studies, whereas human cohort studies more generally indicate diagnostic, prognostic, or stratification potential. NcRNAs listed as biomarkers should therefore not be regarded as validated causal regulators unless they are supported by functional perturbation, disease-model validation, and clinical association.

**TABLE 4:** Diagnostic and prognostic biomarkers in CVDs via ncRNAs.

Biomarker	Type (miRNA/lncRNA/circRNA)	Disease ASSOciation	Clinical use (Diagnosis/prognosis)	References
miR-206	miRNA	MIRI	Potential diagnostic/prognostic biomarker	(Zhai et al., 2017)
miR-133a	miRNA	Perioperative myocardial injury (PMI)	Preoperative prediction and early diagnosis	(Zhou et al., 2022c)
miR-133 b	miRNA	VMC	Disease severity assessment and diagnosis	(Zhang et al., 2017b)
miR-20b-5p	miRNA	AF	Postoperative recurrence prediction and assessment	(Harada et al., 2023)
AK006774	lncRNA	MI	Potential diagnostic/therapeutic target	(Nie et al., 2021)
miR-132–3p	miRNA	Chronic ischemic HF	Prognosis and treatment response biomarker	(Täubel et al., 2021)
miR-483–5p	miRNA	AS/Post-MI	Prognostic biomarker	(Rehberger Likozar et al., 2025)
miR-208b-3p	miRNA	ACS	Prognostic biomarker	(Huang et al., 2023)
LncRNA TUG1	lncRNA	MI	Prognostic biomarker	(Dang et al., 2023)
miR-365–3p	miRNA	CAD (PCI post)	Diagnostic biomarker	(Chen et al., 2019)
LncPPARδ	lncRNA	CAD	Diagnostic biomarker	(Cai et al., 2016)
miR-126	miRNA	HFpEF	Prognostic biomarker	(Jin et al., 2021)
miR-423–5p	miRNA	Hypertensive HF	Prognostic biomarker	(Huang et al., 2022a)
miR-21	miRNA	DCM	Diagnostic biomarker	(Tao et al., 2020)
LIPCAR	lncRNA	CHF	Prognostic biomarker	(Meessen et al., 2021)
miR-132	miRNA	CHF	Prognostic biomarker	(Masson et al., 2018)
miR-126	miRNA	Ischemic heart disease	Potential prognostic biomarker	(Traxler et al., 2023)
miR-133	miRNA	Non-ST segment elevation myocardial infarction (NSTEMI)	Diagnostic for NSTEMI	(Biener et al., 2021)
miR-126	miRNA	CAD	Prognostic for inflammation	(Wang et al., 2016a)
miR-21	miRNA	Myocardial ischemia injury	Prognostic for myocardial protection	(Bartman et al., 2017)
miR-199a-5p	miRNA	HTN/Early CVD	Prognostic for blood pressure changes	(Lynch et al., 2020)
miR-124–3p	miRNA	Cardiac arrest	Prognostic for neurological outcomes	(Devaux et al., 2016)
miR-126–3p	miRNA	Cardiovascular metabolic health	Diagnostic risk prediction	(Piacquadio et al., 2024)
miR-574–5p	miRNA	Cardiac arrest	Prognostic evaluation	(Boileau et al., 2019)
miR-125 b	miRNA	AMI	Prognostic assessment	(Gasecka et al., 2024)
miR-145	miRNA	AMI	Prognostic HF risk	(Zhang et al., 2017a)
SNHG8	lncRNA	MI	Diagnostic for myocardial injury	(Zhang and Bian, 2020)
PCAT19	lncRNA	CAD	Diagnostic for AMI onset	(Zhou et al., 2024a)
hsa_circRNA_0001599	circRNA	Large artery AS ischemic stroke (LAA-stroke)	Diagnostic biomarker	(Li et al., 2021a)
MEG3	lncRNA	VMC	Prognostic biomarker (related to heart function and inflammation)	(Xue et al., 2020)

Although these ncRNAs have good potential as molecular markers in precision phenotyping of CVD and risk prediction, more independent clinic-based studies are required to validate them empirically. While the dynamic expression and accessibility of these ncRNAs offer obvious advantages, it is important to acknowledge that the key regulatory roles of miRNAs, lncRNAs and circRNA are highly context-dependent and that validation across studies is relatively low. Much of the evidence, particularly for circulating miRNA biomarkers, is derived from relatively small cohorts and non-standardized analytic methodologies, with limited longitudinal validation (Searles, 2024). The stability of ncRNAs in biological fluids is both advantageous and limiting with respect to signal, interference or originating from diverse cell populations. Recent advances undoubtedly offer analytical sensitivity though the key challenge will be developing a mechanistic and quantifiable association with ncRNA expression and relevant underlying disease activity.

### 3.2 Advances in emerging detection technologies

The development of molecular diagnostic tools and nanosensing technologies has enabled ncRNA detection to evolve from polymerase chain reaction (PCR)-based methods to high-signal-to-noise readouts in electrochemical, photoelectrochemical, and multimodal formats. These technologies offer improved analytical performance and faster turnaround times, thereby expediting the clinical translation of ncRNA quantitation.

The remarkable stability of circRNAs encourages their use as blood-borne biomarkers. Circulating circRNAs are useful biomarkers for atherogenic vascular disease, with levels reflecting inflammation, injury, or lesion instability at onset (Northoff et al., 2025). As a candidate marker for acute and chronic cardiovascular conditions, miR-483–5p participates in healing injury and disease progression (Volodko et al., 2023). MiR-3135b, miR-3908, and miR-5571–5p can distinguish heart failure with reduced ejection fraction (HFrEF) from HFpEF, providing an approach to phenotype stratification in HF (Chen F. et al., 2018). MiR-22–3p shows high diagnostic specificity for CAD and acts as a molecular adjunct to conventional tests (Zhang M. et al., 2022).

NcRNAs also serve as diagnostic tools in children. Expression of exosomal miR-146a-5p, miR-23a-3p, and miR-27a-3p is highly increased in children with acute myocarditis, with miR-146a-5p showing the best diagnostic value (Zhang X. et al., 2023). Regarding clopidogrel resistance in CAD, the expression of hsa_circ_0057714 and hsa_circ_0076957 can predict responses to antiplatelet therapy. Mechanistically, hsa_circ_0076957 mediates COL19A1 expression via miR-4512 (Xu H. et al., 2025). Additionally, miRNA profiling reveals mechanistic pathways not affected by present therapies but important to patients with heterogeneous genetic landscapes (Kanuri et al., 2018). Let-7i-3p enhances the diagnostic accuracy of coronary artery aneurysm (CAA), especially in combination with ultrasound measurement (Wang et al., 2019). Besides, hsa_circ_0043621 shows diagnostic value in carotid AS associated with NLRP3-mediated inflammation (Yan et al., 2024).

Detection limits have been reduced by methodological developments. A fluorescence resonance energy transfer (FRET)-based miR-208a sensor decreases assay time to 5 min with a detection limit of 380 pM (Zhang S. et al., 2025). Metalene-induced electrochemiluminescence (ECL) sensors achieve early detection of coronary artery calcification (CAC) with an area under the curve (AUC) of 0.967, surpassing common PCR (Cui et al., 2025). Moreover, a photo-nanozyme photoelectrochemical biosensor detects miR-133a with a limit of detection (LOD) of 0.003 fM (Li HJ. et al., 2025), and CND ECL systems directly sense miR-21 levels in the serum of HF patients without RNA extraction (Gutiérrez-Gálvez et al., 2021).

Current ncRNA detection methods, particularly extracellular miRNA quantification platforms, vary greatly in analytical sensitivity, dynamic range, specificity, and clinical practicality (Godoy et al., 2019). These platform-dependent features affect the stability and interpretability of biomarker measurements. An optimal choice of platform is therefore important for biomarker discovery, clinical validation, and subsequent clinical use across different CVD contexts. Table 5 gives an overview of relevant methodological parameters and indications of current platforms for CVD evaluation.

**TABLE 5:** Comparative overview of ncRNA detection technologies.

Detection method	Advantages	Limitations	Typical detection limit	Linear range	Sample type	References
Non-enzymatic surface-enhanced Raman scattering (SERS) biosensor (CHA-HCR amplification)	High sensitivity, specificity, rapid detection	Not widely validated clinically	5.13 fM	10 fM–1.0 nM	Red blood cell samples	(Chen et al., 2024c)
TRHDA-mediated Cas12a method	High sensitivity, high specificity, single-base resolution	Requires precise DNA hairpin design and optimization	13.9–32.4 pM	50 pM–15 nM	*In vitro* miRNA detection + cell imaging	(Zhao et al., 2024)
NEM-IR-RCA (cut enzyme interference reduction rolling circle amplification)	Ultra-sensitive, high specificity, no reverse transcription, isothermal detection	Dependent on synthetic probe systems and signal amplification design	0.0095 fM	0.05–100 fM	Cancer cells, mouse serum	(Chen et al., 2022c)
Microfluidic miRNA detection based on DNA locking/unlocking states	Fast assembly (2 min), amplifiable signals, applicable to complex samples	Dependent on enzyme cleavage and magnetic separation, requires microfluidic devices	2.01 pM (S/N = 3)	3.2–570 pM	Serum, urine, saliva	(Liu et al., 2022)
SOI-NW label-free electrochemical biosensor	High sensitivity, no amplification or labeling required	High gate pressure, small sample size	1.1 × 10^−16^ M	1.1 × 10^−16^–1.1 × 10^−14^ M	Buffer, plasma samples	(Ivanov et al., 2022)
Fluorescent biosensor based on RCA	High sensitivity, high selectivity	Requires multiple primer designs	0.06 pM	1 pM–100 nM	Liver cancer cells, whole blood	(Yao et al., 2023)
Light-induced ATRP electrochemical biosensor	High sensitivity, low cost, anti-interference	Requires light reaction conditions	1.35 fM	10 fM–1 nM	Serum samples	(Yu et al., 2023b)
Self-amplified ECL electrochemical biosensor	Ultra-sensitive, reproducible	Complex reaction system	0.03 fM	0.1 fM–1 nM	*In vitro* simulated samples or breast cancer-related samples	(Sun et al., 2023)
[Ru (bpy)3]^2+^/CNDs ECL biosensor	High sensitivity, no amplification required	Needs prolonged probe fixation	0.721 fM	2.34 fM–100 pM	Human serum samples	(Gutiérrez-Gálvez et al., 2021)
Hairpin probe-assisted ICSDP microfluidic detection	No RNA extraction, sensitive, simple	Requires temperature control and enzyme reactions	34 fmol L^-1^	0–0.5 pmol L^-1^	Synovial fluid samples	(Bellassai et al., 2022)
SGGT-based label-free electrochemical sensing	No extraction/amplification, real-time detection	Limited clinical sample volume	10^−20^ M	10^−20^–10^−12^ M	Serum samples	(Deng et al., 2023)
PtμE/MoS2/Au microelectrode SWV detection	High sensitivity, good selectivity, detects whole blood	Requires longer incubation time (∼0.5 h)	1.6 × 10^−12^ M	10^−11^–10^−8^ M	Blood samples (20 μL)	(Zhai et al., 2023)
SiO2 chip-based enrichment detection	Efficient exosome capture, high sensitivity	Requires smaller sample volumes	10,000 particles/mL	10 to 10,000,000 particles/mL	Clinical plasma, HCC patient plasma	(Yi et al., 2024)
Integrated SAW-IEM microfluidic platform	No PCR, rapid, high sensitivity	Detects only single-target miRNA	1 pM	1 pM–1 nM	Plasma, serum	(Ramshani et al., 2019)
EZ-READ platform	Direct RNA detection, no amplification	Currently validated only in GBM samples	9 copies	Wide range linear quantification	Plasma and tissue samples	(Zhang et al., 2023c)
HRP-embedded DNA hydrogel photoelectrochemical method	No specialized instruments, low cost	Higher detection limit	7.8 nM	0.5–4.0 µM	HeLa cell lysate	(Liu X. et al., 2021)
BiMW nanophotonic biosensor	Label-free, real-time detection	Early sample limitations	25 pM	0.5–100 nM	Human plasma samples	(Calvo-Lozano et al., 2022)
U-shaped fiber-optic miRNA biosensor	High sensitivity, strong specificity	Complex fiber preparation	0.0133 ng/mL; (0.5 nM)	0.05–100 nM	Standard samples, liver cancer cells	(Wen et al., 2020)
ExoAgo dual-amplification system	Dual amplification, highly sensitive	Requires high-temperature reaction (85 °C)	12.2 pM	10 pM–1 μM	Diluted plasma samples	(Wang et al., 2023b)
PECL-clustered regularly interspaced short palindromic repeats (CRISPR) electrochemical luminescence method	Ultra-sensitive, single-base resolution	Needs multi-enzyme complex systems	1 × 10^−15^ M	1 fM–100 pM	Cell extracts, lysates	(Zhou et al., 2020c)
Graphene/PBSE electrochemical biosensor	High sensitivity, specificity, fast detection	Stability needs improvement	10^−11^ M (buffer), 10^−10^ M (serum)	10^−11^–10^−6^ M (buffer), 10^−11^–10^−7^ M (serum)	Buffer, diluted serum samples	(Nagdeve et al., 2025)
DSN cycle amplification + magneto-optical detection	Highly sensitive, multiplex detection	Requires specific equipment	4.8 fM	10 fM–10 nM	Serum	(Tian et al., 2017)
DSN amplification with PGM detection	High sensitivity, low cost, portable	Longer reaction time	1.8 pM	10–200 pM	Urine samples, mouse urine	(Huang et al., 2020d)
Electrochemical biosensor (streptavidin-modified SPCE)	Highly sensitive, fast, and selective	Multiple surface modifications, relatively complex operation	0.33 fM	100 nM–100 fM	Plant extract samples	(Nehra et al., 2022)
Magnetic separation-bio/color/electrochemical dual-mode detection	No amplification, highly specific, low cost	Non-specific adsorption, many steps	1 fM	1 fM–1 nM	Human plasma, ovarian cancer cells	(Soda et al., 2020)
Au NCs/MWCNT-NH2 electrochemical biosensor	High sensitivity, low cost, easy operation	Detects only a single lncRNA	42.8 fM	0.01 pM–10 nM	Human serum samples	(Chen et al., 2021e)
Enzyme-free self-assembled nanobiosensor	High specificity, stability, *in-situ* detection	Lack of widespread validation	1 fM	1 fM–10 pM	Cardiomyocyte models, plasma, serum, tissue	(Li et al., 2024c)
GQDs-PMO-modified RGO-FET sensor	High sensitivity, specificity, stability, discriminates cancer samples	Blood sample interference affects accuracy	85 a.m.	100 aM–1 nM	Breast cancer patient plasma exosomes	(Li et al., 2022d)
CRISPR/Cas13a + gold nanoparticle array	Ultra-sensitive, no RNA degradation, preserves EV integrity	Requires precise control for fusion and cancer-specific EV discrimination	2.5 × 10^1^ EV particles/μL	10–10^6^ EV particles/μL	Plasma, 3D VTS, cell culture	(Chen et al., 2025b)
Thiol probe and PEI-AuNP crosslinked	Sensitive, simple, low-cost, label-free	Requires optimization of nanoparticle ratio and conditions	100 a.m.	10^2–10^5 aM	*In vitro* synthesized miRNA standard solutions	(Hakimian et al., 2018)

While the field is progressing toward high sensitivity and multidimensional signal integration, a disconnect remains between methodologic novelty and biological interpretation. Assay time and accuracy have been improved in FRET, electrochemical, and photoelectrochemical systems. However, standardization and reproducibility remain major barriers, especially for miRNA-based biomarker assays (DE Gonzalo‐Calvo et al., 2022). Suboptimal performance between platforms (variation in sensitivity, specificity, and dynamic range) hinders inter-study comparisons and knowledge translation. Moreover, increased precision will not solve fundamental issues of signal heterogeneity and mixed cell population origins. Without biological validation, sensitive detection technologies alone are insufficient to establish ncRNAs as reliable clinical biomarkers.

### 3.3 Challenges and prospects for clinical translation

Mechanistic understanding and biomarker discovery for ncRNAs are relatively advanced, but clinical translation is still in its infancy. Significant hurdles must be overcome, such as safety testing, dose finding, assessment of long-term effects, and managing interindividual variability. In human clinical trials, the miRNA inhibitor CDR132 L selectively suppressed miR-132 and reduced pathologic ventricular remodeling in patients with HF; safety and efficacy data support moving to larger clinical trials (Täubel et al., 2021). In peripheral arterial disease, miR-126 is increased by stimulation, leading to augmented angiogenesis in the ischemic leg, mediated through inhibition of PI3KR2 and activation of the VEGF pathway, with improvement in local perfusion (Da Silva et al., 2023).

When it comes to antiplatelet therapy, the drug-specific influence on miRNA expression has clinical importance. Circulating miR-125 b is reduced by ticagrelor in acute MI patients, and high miR-125 b levels in clopidogrel-treated patients are correlated with recurrent thrombosis and adverse clinical events (Gasecka et al., 2024). In terms of prognostic stratification, exosomal miR-208b-3p and miR-143–3p are associated with sudden cardiac death in the setting of acute coronary syndrome (ACS), indicating possible application in forensic and clinical practice (Huang et al., 2023). However, in patients assessed for suspected ACS, miR-21–5p and miR-122–5p did not add value to prognosis over hscTnT (Biener et al., 2022).

In inflammatory CVDs, coordinated miRNA regulation determines cytokine activity in carotid atherosclerosis (CAS). The inhibitory role of miR-146a implies its participation in disease modulation despite incomplete knowledge of its interactome (Huang et al., 2020a). In the setting of non-ST elevation acute coronary syndrome (NSTE-ACS), several miRNA-mediated genomic pathways contributing to disease pathogenesis have been uncovered, representing a blueprint for future mechanistically-based prognostic models (Kwee et al., 2019).

From a therapeutic perspective, it is known that miR-21 functions downstream of Per2 to regulate the metabolic response to light, suggesting potential crosstalk between environmental cues and miRNA pathways (Bartman et al., 2017). In terms of risk prediction for HF readmission, low levels of circulating miR-132 provide incremental prognostic information beyond traditional clinical variables, supporting its potential utility in therapeutic strategy (Masson et al., 2018). In HFpEF, exercise rehabilitation raises plasma miR-126, which is associated with improved cardiac function and exercise capacity (Jin et al., 2021).

The clinical translation of ncRNAs is being developed from the concept proof to a comprehensive translational pipeline. This evolution is predicated on three major factors: biological specificity and structural stability required for clinical application; increasingly sensitive detection techniques enabling quantification feasibility; and emerging therapeutic platforms such as targeted inhibitors and exosome-based delivery that signal the dawn of interventional application (Zhang et al., 2026b). Together, these advances blur the difference between molecular markers and therapeutic targets. They establish a basis for ncRNA-targeted personalized cardiovascular medicine and are in line with the overall mechanistic and precision-intervention concepts outlined in this work.

That said, translation to the clinic is not without challenges. While agents such as CDR132 L have provided early clinical evidence of target engagement and exploratory signals of reverse remodeling in HF (Täubel et al., 2021), most miRNA-targeted candidates still face outstanding questions related to dosage, tissue-specific delivery capacity, delivery efficiency, immune responses, off-target effects, and long-term safety (Saenz-pipaon and Dichek, 2023). Notable disease- and model-specific miRNA effects further highlight mechanistic complexity and the difficulty of extrapolating preclinical results to human CVD. Additionally, biomarker changes or improved target detection should not be equated with prognostic significance or clinical causality without outcome-level validation. The most significant hurdle is thus converting expression correlations into mechanistically confirmed and clinically actionable treatments.

## 4 ncRNAs in CVD

Playing a role throughout the evolution of CVD, ncRNAs regulate significant pathological switches involving endothelial dysfunction, lipid accumulation, inflammation, myocardial remodeling, and vascular structure alterations. Distinct classes of ncRNA make up interacting regulatory circuitries along common pathogenic axes, controlling disease progression and offering potential therapeutic targets. After introducing their biological functions and biomarker potential, this section discusses the impact of ncRNAs in major cardiovascular pathologies, specifically AS, HF pathophysiology, and HTN-related vascular remodeling.

### 4.1 ncRNAs in AS

Atherosclerotic development is associated with endothelial injury, the transition of VSMC phenotype, immune cell infiltration, and changes in plaque stability. During these shifts, ncRNAs create a web of interacting axes that combine metabolic stress and perturbed vascular responses. Under conditions of endothelial injury and metabolic disturbance, circ_0026218 suppresses oxidized LDL (oxLDL)-induced damage in human umbilical vein endothelial cells (HUVECs) by sponging miR-338–3p to increase SIRT6. This mechanism indicates that circRNAs may retain deacetylation pathways to respond against early endothelial dysfunction (Yang L. et al., 2023). MiR-132/212-enriched exosomes from adipose tissue are implicated in AS. They augment palmitate-induced endothelial apoptosis by inhibiting Gα12 and accelerate PDGF-BB-stimulated smooth muscle cell proliferation and migration through the regulation of PTEN signaling. Together, these mechanisms form a single axis that connects metabolic stress with vascular remodeling (Guo et al., 2024). Melatonin suppresses these effects by downregulating exosomal miR-132/212, and this finding indicates that the regulation of exosome-delivered miRNA may be a cellular strategy for preventing metabolic vascular injury (Guo et al., 2024). The circRNA hsa_circ_0001445 is decreased during AS; coronary smooth muscle cells release it into the blood, where it improves the diagnostic value of computed tomography (CT) angiography in suspected stable CAD (Vilades et al., 2020).

Regarding apoptotic and inflammatory regulation, miR-21 knockout causes the upregulation of Xaf1 in atherosclerotic aortas and perturbs the circadian expression of this target. This suggests that its antiapoptotic effects on the cardiovascular system are largely dependent upon this direct target (Schober et al., 2021). MiR-202–5p levels promote macrophage apoptosis, thereby promoting necrotic core formation and the occurrence of vulnerable plaques (Xu et al., 2023). CircANRIL, an autophagy-associated circRNA that regulates ribosomal RNA maturation, confers protection against AS. This result indicates that the circularization of long ncRNAs changes their functions and provides a structural basis for RNA drug design (Holdt et al., 2016).

Within the immune-inflammatory axis and plaque stability, the absence of lncNEXN promotes accelerated atherosclerotic lesion development, involving enhanced macrophage recruitment in combination with increased expression of adhesion molecules and inflammatory mediators. In contrast, upregulation of NEXN inhibits the development of disease (Hu et al., 2019). Exosomes from IgE-stimulated mast cells contain circCDR1as, leading to endothelial dysfunction and atherogenesis. This discovery links allergic inflammation with vascular pathology and implies that ncRNAs from mast cells may provide an independent risk factor in asthma or other atopic diseases (Yang H. et al., 2024). CircZBTB46 modulates the PTEN/AKT/mTOR pathway in CAD through interaction with hnRNPA2B1 and markedly influences disease progression (Fu et al., 2023). MiR-494–3p activates Wnt signaling to inhibit M1 and accelerate M2 macrophage polarization, leading to the attenuation of plaque inflammatory burden and improved stability (Van ingen et al., 2021). The inhibition of lncRNA HCG11 suppresses pyroptosis and inflammation caused by ox-LDL by upregulating the miR-224–3p/JAK1 axis, providing additional evidence for the importance of pyroptotic pathways in atherosclerotic arterial damage (Zhou and Song, 2023).

In conclusion, miRNAs, lncRNAs, and circRNAs constitute an interconnected regulatory network in endothelial injury, smooth muscle cell remodeling, immune polarization, and programmed inflammatory cell death. These molecules act as both drivers of disease and members of intrinsic protective systems. As a result, they represent candidate targets for anti-atherosclerotic therapy (QI et al., 2024). The relationship among ncRNAs, endothelial dysfunction, immune remodeling, and vascular structural evolution in AS is summarized in Figure 4 as three core regulatory axes.

**FIGURE 4:**
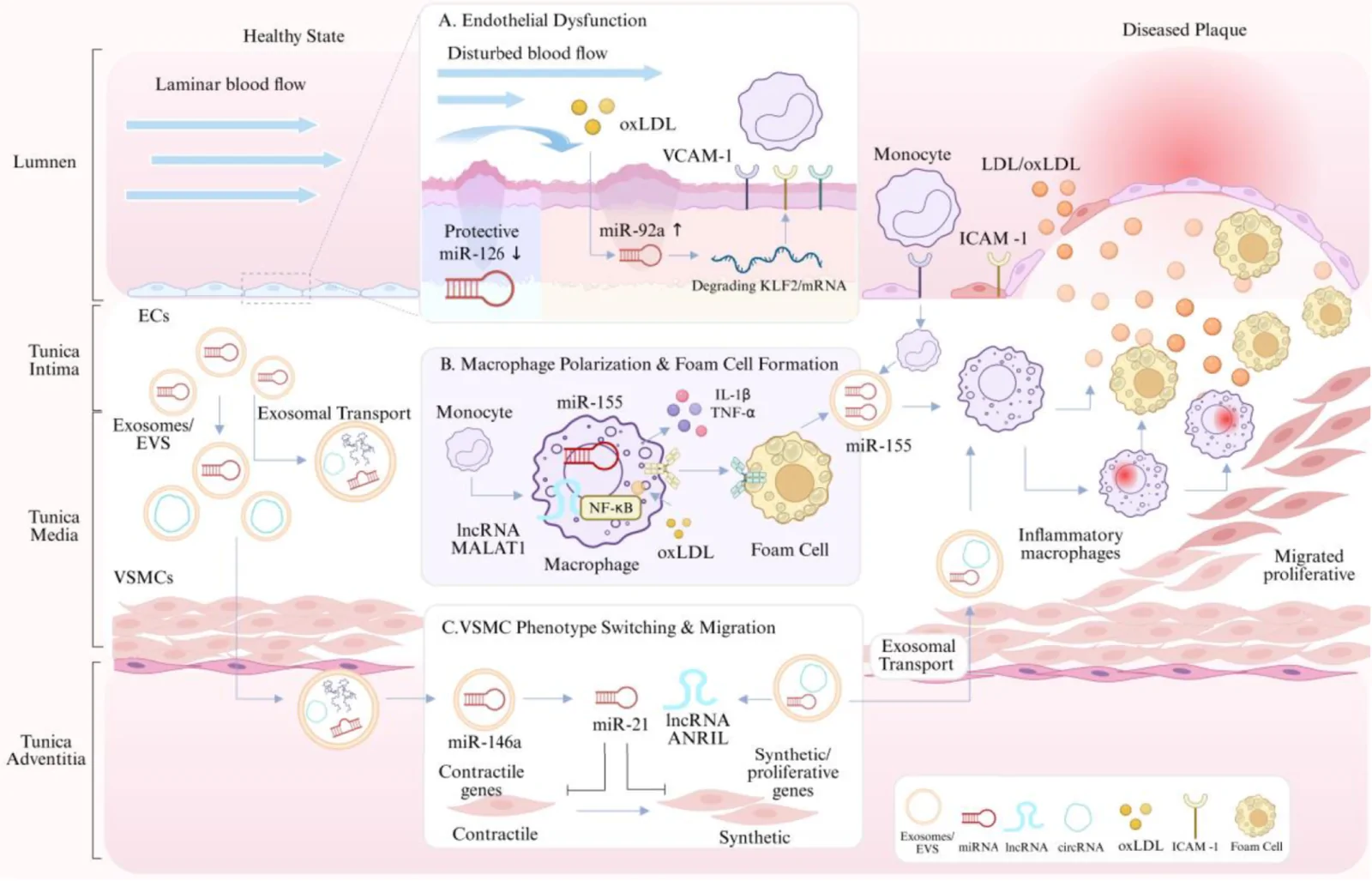
Mechanistic roles of ncRNAs in atherosclerosis. This figure illustrates how ncRNAs coordinate pathological processes across vascular cell types during atherosclerosis (AS) progression. **(A)** Disturbed blood flow and oxidized low-density lipoprotein (oxLDL) disrupt endothelial homeostasis, accompanied by decreased protective miR-126 and increased miR-92a expression. These changes impair endothelial protective signaling, including KLF2-related regulation, and promote the expression of adhesion molecules such as VCAM-1 and ICAM-1, thereby facilitating monocyte adhesion and transendothelial migration. **(B)** Infiltrating monocytes differentiate into macrophages, where oxLDL uptake and ncRNA-mediated regulation contribute to inflammatory activation and foam cell formation. lncRNA MALAT1 and miR-155 participate in the regulation of NF-κB-associated inflammatory signaling and cytokine production, including IL-1β and TNF-α, thereby promoting macrophage activation and local inflammation. **(C)** In vascular smooth muscle cells (VSMCs), exosome-mediated ncRNA transfer and regulatory molecules including miR-146a, miR-21, and lncRNA ANRIL influence the balance between contractile and synthetic phenotypes, promoting VSMC migration and proliferation toward atherosclerotic lesions. Together, these ncRNA-mediated processes integrate endothelial dysfunction, macrophage activation and foam cell formation, and VSMC phenotypic remodeling to promote atherosclerotic plaque progression.

The ncRNAs act as an important molecular layer in the pathophysiology of AS, HF, and HTN, while their biological effects show both activation and inhibition. Some species play a protective role through the downregulation of inflammation or promoting repair of the endothelial lining, whilst others promote immune polarization and structural change. This establishes a biological setting for the presence of pro-pathogenic and anti-pathogenic signals. The overlap of distinct RNA species in common pathogenic processes reflects a complex and redundant regulatory network (Zhang W. et al., 2025; Zhu et al., 2021). Variability between experimental models and population heterogeneity results in a lack of comparability, while contemporary pathway analyses focus on single-dimensional model structures. A significant issue is how to separate genuine regulatory hubs from noise at the systemic scale. This is a crucial step to ascertain whether ncRNAs move from being observed biological markers toward integrative pathophysiological models in CVD.

### 4.2 Interplay between HF and ncRNAs

The key mechanisms involving HF encompass abnormal energy metabolism, mitochondrial impairment, myocardial fibrosis, microvascular rarefaction; and networks of ncRNAs are orchestrating conductors in these aspects. For energy metabolism and mitochondrial homeostasis, circSamd4 enhances the translocation of Vcp into mitochondria and decreases oxidative stress to maintain the normal mitochondrial dynamics in addition to down-regulating Vdac1 for avoiding the onset of mitochondrial permeability transition pore (mPTP). These combined effects reduce post-MI remodeling and prevent slow progression to chronic heart failure (CHF) (Zheng et al., 2022). CircIGF1R regulates the proliferation of fibroblasts through inhibition of glycolysis metabolic reprogramming. Thus, ectopic expression of circIGF1R in HF patient-derived fibroblasts causes a remarkable anti-fibrotic effect (Schmidt et al., 2025). CircSnap47 knockdown mitigates oxygen-glucose deprivation (OGD)-induced injury in cardiomyocytes via the miR-223–3p/MAPK pathway. This capability limits the augmentation of maladaptive remodeling signals that are responsible for the progression of HF (Wang Y. et al., 2023).

As for fibrosis and structural remodeling, separate regulatory programs are apparent. The direction for miR-132 in treatment is inclined to be inhibitory; however, evidence also shows that upregulated levels of miR-132 ease the fibrotic burden and improve cardiac function during HF via repressing PTEN and blunting PI3K/Akt signaling (Wang G. et al., 2020). LncFoxo6os directly interacts with MYBPC3 and mediates its association with PKC-α to induce phosphorylation. This mechanism is critically important for preserving myocardial contractility and retarding the advance of HF (Sheng et al., 2025). In contrast, lncMALAT1 worsens lipid metabolism and pathological injury by competing for miR-532–3p and promoting the expression of LDLR (Zhao et al., 2021). These molecules constitute a lncRNA axis implicated in the myocardial structural remodeling and biomechanical properties of failing hearts. From a microvascular function and tissue perfusion perspective, miR-665 inhibits myocardial infarct border zone angiogenesis and is directly involved in the progression to HF, thereby indicating that reconstruction of microvascular density and function can be a therapeutic strategy for decreasing cardiac dysfunction (Fan et al., 2018). MiR-221–3p is inhibitory to endothelial angiogenesis via regulation of HIF-1α. Its blockade enhances cardiac function in transverse aortic constriction (TAC) mice and may serve as a prognostic marker (Li et al., 2021c). MiR-128 enhances HF and hypertrophic remodeling by promoting Wnt1/β-catenin pathway activity in an Axin1-dependent manner (Li JY. et al., 2021). When combined with N-terminal pro-B-type natriuretic peptide (NT-proBNP), miR-208a increases the diagnostic accuracy for HFrEF and thereby underlines the value of integrating ncRNAs with established biomarkers in clinical diagnostics (Li DM. et al., 2021).

Collectively, ncRNAs involved in HF, particularly HFpEF, not only contribute to the metabolic and mitochondrial abnormalities that drive early injury but also influence fibrotic remodeling, microvascular adaptation, and the later progression to cardiac dysfunction that characterizes the disease. Their differential expression patterns are associated with specific clinical phenotypes and may support molecular classification and stratification for targeted therapy (Jalink et al., 2024). Figure 5 depicts the multifaceted regulatory axes among which ncRNAs integrate cardiomyocyte injury, fibrotic remodeling, and immune activation, highlighting their pivotal system-level position in HF development.

**FIGURE 5:**
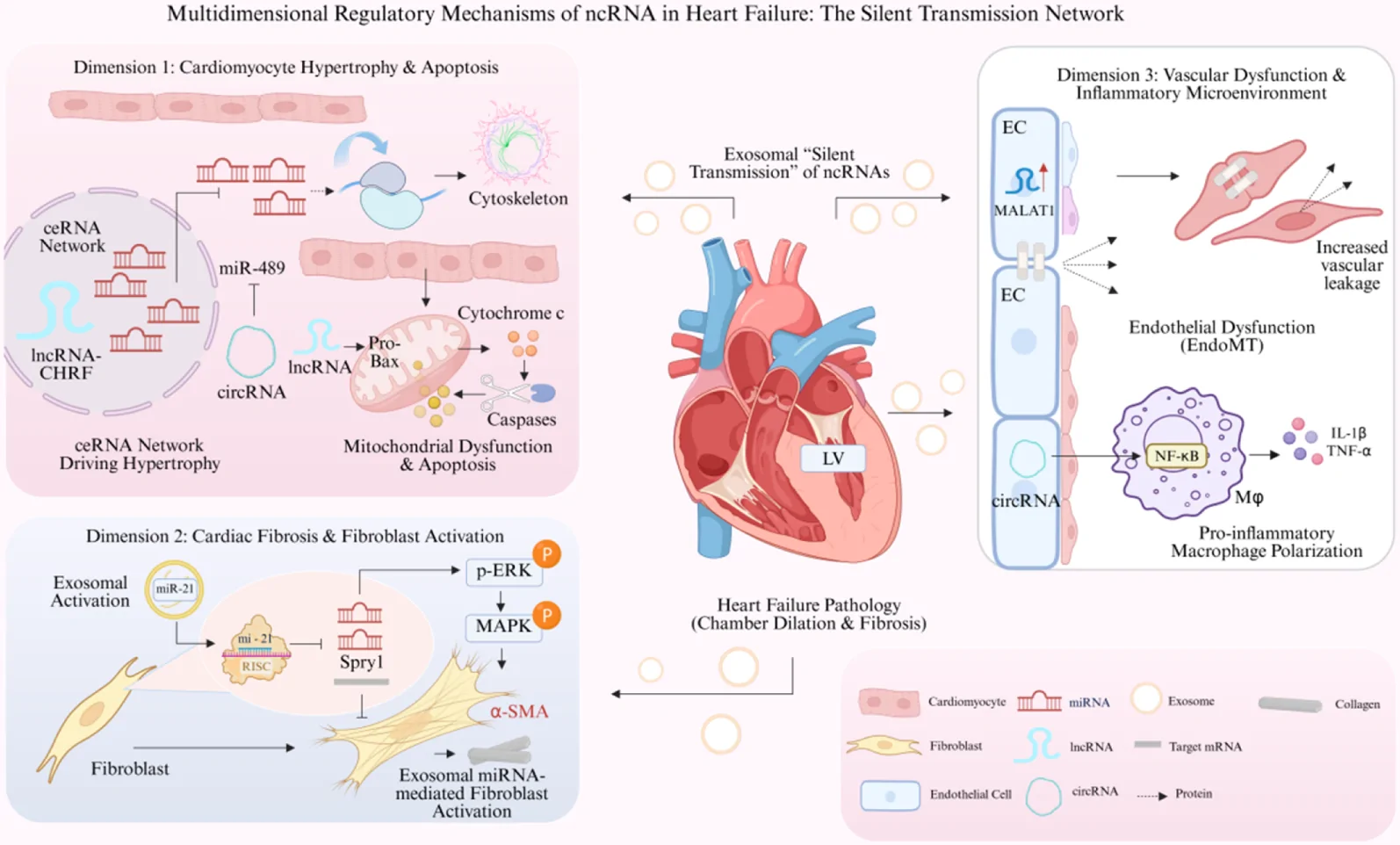
Multidimensional mechanisms through which ncRNAs regulate HF. This figure demonstrates the multilayered regulatory networks regulated by ncRNAs during structural remodeling and inflammation in HF. Within cardiomyocytes, lncRNAs and circRNAs form ceRNA interaction modules that control key nodes involved in cytoskeletal remodeling, mitochondrial injury, and apoptosis, which drives molecular programs toward hypertrophy and cell loss. In fibroblasts, the uptake of exosomal miR-21 derived from cardiomyocytes alters the Spry1/MAPK/ERK axis, promoting myofibroblast differentiation and collagen accumulation, and accelerates interstitial remodeling. At the vascular and immune interface, endothelial MALAT1 and specific circRNAs contribute to endothelial-to-mesenchymal transition (EndoMT), increased permeability, and the activation of macrophage NF-κB signaling, collectively fostering a pro-inflammatory environment. The integration of these signals across cell types establishes ncRNAs as critical regulators that drive the progression of HF through coordinated pathways involving cardiomyocyte injury, fibrotic expansion, and immune activation.

NcRNAs serve as an important layer of regulatory molecules within the molecular pathogenesis of HF, and their functional roles are highly context-dependent. Some molecules, like circSamd4 and lncFoxo6os, exert cardioprotective effects by maintaining metabolic remodeling and mitochondrial integrity. Others, such as MALAT1 and miR-128, are involved in pathological events, including lipid disorders and fibrosis. It is worth noting that molecules such as miR-132 display intricate context-specific effects in diverse studies. Their signaling effects may diverge among experimental models depending on cellular context and metabolic state. Most existing studies still focus on isolated pathways, with limited system-level integration and systematic validation (Jalink et al., 2024; Ao et al., 2023).

### 4.3 Advances in ncRNA research in HTN

Endothelial dysfunction, vascular smooth muscle proliferation, and progressive remodeling of vessels are promoting factors for hemodynamic disorders such as systemic HTN and pulmonary arterial hypertension (PAH). NcRNAs serve as central regulators in these processes by sensing mechanical overload, neurohumoral activation, and cell-intrinsic programs within the heart.

In systemic HTN, miR-483–3p is involved in disease initiation, mediating endothelial protection and modulation of endothelial function, thus highlighting its role as a therapeutic target for hypertensive-related cardiovascular injury (Shang et al., 2022). LncGAS5 exerts two-way modulation on the function of endothelial cells and VSMCs through β-catenin signaling, leading to potential molecular mechanisms for gene-based and chemotherapeutic treatment in HTN (Wang YN. et al., 2016). Dihydromyricetin ameliorates endothelial dysfunction and improves the atheroprotective effect of hyper-HDL in spontaneously hypertensive rats through miR-24-induced activation of the PI3K/AKT/eNOS pathway. This result gives a miRNA-based mechanistic explanation for the blood pressure-lowering effects of this traditional small molecule (Yang X. et al., 2024). In epidemiological studies, upregulation of hsa_circ_0105,015 is positively related to the risk for essential HTN; corresponding to a decrease in the level of circulatory hsa-miR-637, this signature may present vascular inflammation and endothelial dysfunction as early pathological markers with diagnostic implications (He et al., 2021). Hsa_circ_0037911 and hsa_mir_637 were also suggested as potential early diagnostic biomarkers for essential HTN when combined (Bao et al., 2019). Hsa_circ_0037909 could potentially regulate serum creatinine or LDL metabolism to influence disease susceptibility (Bao et al., 2019).

In PAH and right ventricular remodeling, ADAR1-mediated m1A modification of circCDK17 enhances VSMC proliferation to facilitate pathogenesis (Zhang J. et al., 2023). CircKrt4, a super-enhancer-related circRNA, promotes the progression of PAH through Pura and Glpk-mediated pulmonary endothelial injury (Ma C. et al., 2023). LncUNC5B-AS1 is a super-enhancer-driven gene that plays an important role in the hypoxia-induced phenotypic switch of pulmonary arterial smooth muscle cells (PASMCs) and has been suggested to inhibit pulmonary vascular remodeling when overexpressed (Zhu et al., 2025). LncFGD5-AS1 and its incorporated micropeptide are therapeutic targets for right ventricular dysfunction in PAH (Chen Y. et al., 2025). High circSSR1 expression increases SSR1 expression, activates the endoplasmic reticulum stress response, and promotes pyroptosis in smooth muscle cells. These events together result in PAH development (Guan et al., 2024). Hypoxia regulates the function of pulmonary microvascular endothelial cells via the miR-17–3p/YTHDF2 axis, which presents a new layer of mechanism in pulmonary hypertension (PH) pathogenesis (Hu X. et al., 2024). In neonatal PAH, hypoxia-induced upregulation of miR-210 in newborn lambs inhibits spontaneous transient outward currents (STOCs), promoting the occurrence of pulmonary HTN (Hu et al., 2025).

These studies show that ncRNAs are constant regulators, covering a spectrum from systemic HTN to PAH, and adult to neonatal populations when it comes to blood pressure control, vascular remodeling, and right ventricular adaptation (Li Z. et al., 2025). As molecular connectors that link hemodynamic load to cellular response programs, they provide new approaches for early identification and directed therapy in HTN and its complications. This further emphasizes the central role of ncRNAs in personalized cardiovascular treatment. Figure 6 summarizes the role of ncRNAs as a central hub responsible for connecting hemodynamic stress with vascular cell remodeling that contributes to the pathologic progression of HTN and PAH.

**FIGURE 6:**
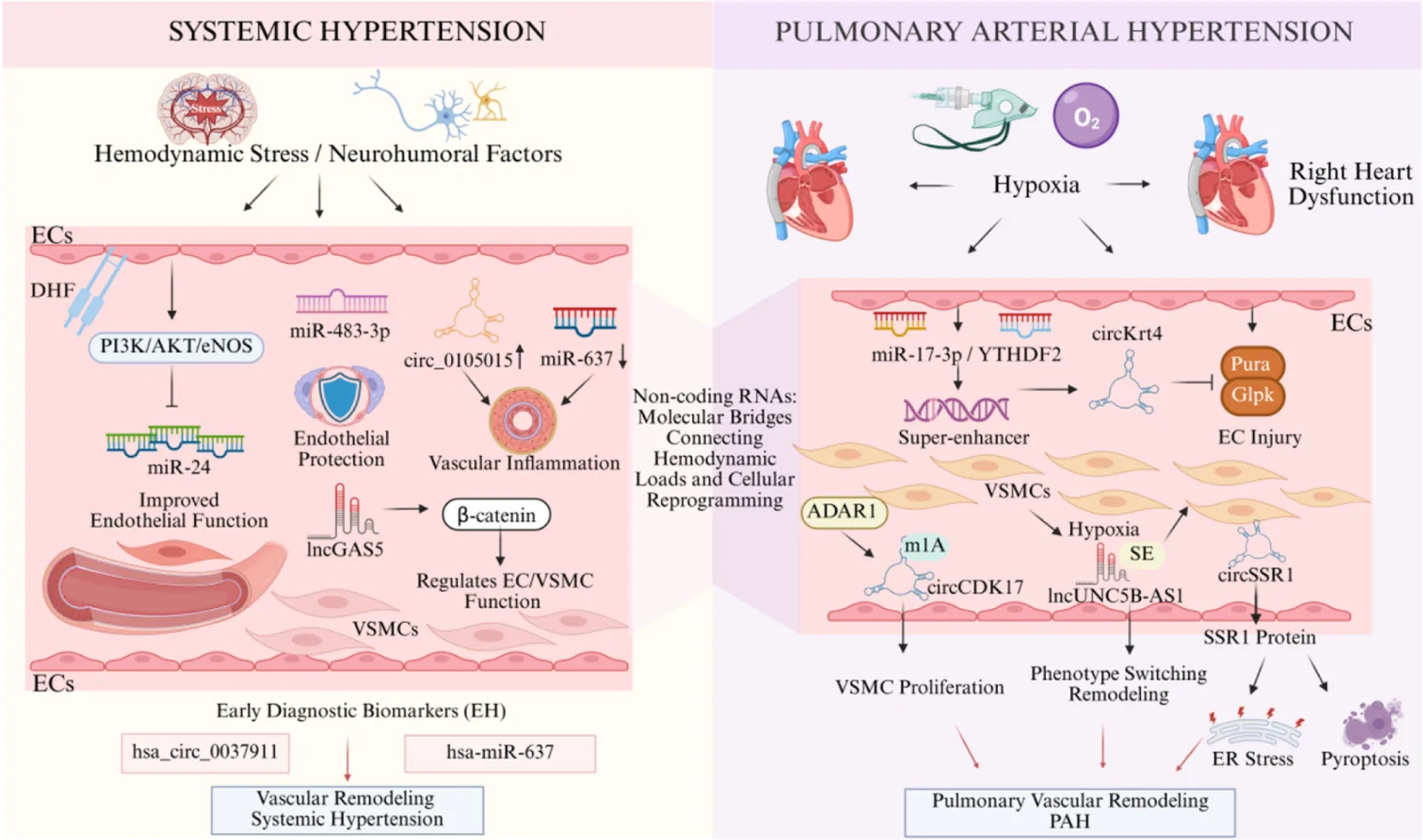
NcRNA-mediated signalling axes in systemic HTN and PAH. This figure depicts the routes of cross cellular and cross pathway regulatory networks formed by ncRNAs in systemic HTN and PAH. In systemic HTN, hemodynamic load and neurohumoral signaling modulate endothelial homeostasis. MiR-24 is associated with PI3K/AKT/eNOS-mediated improvement of endothelial function, whereas miR-483-3p contributes to endothelial protection. Conversely, circ_0105,015, miR-637, and lncGAS5 modulate inflammatory programs and coupling between endothelial and smooth muscle cells, thereby initiating early vascular remodeling. Within the pulmonary arterial compartment, pathways induced by hypoxia, specifically those involving the miR-17–3p/YTHDF2 axis, circKrt4, and associated protein complexes, worsen endothelial injury. Molecules such as lncUNC5B-AS1 and circCDK17 participate in m^1^A dependent regulation and super enhancer activity to promote smooth muscle cell proliferation and phenotypic reprogramming, linking these processes to downstream events such as endoplasmic reticulum stress and pyroptosis. Overall, the framework demonstrates ncRNAs as critical signaling hubs that integrate mechanical stress with vascular cell remodeling, thereby regulating the systemic progression of HTN and PAH.

The ncRNAs represent critical nodes in the link between hemodynamic stress and vascular remodeling, although their functional aspects are highly dual. Molecules like miR-483–3p and lncGAS5 contribute to vasoprotection of endothelial function, while circCDK17 and circSSR1 trigger pathological proliferation and inflammatory pathways (Zhang J. et al., 2023; De Rosa et al., 2022). The disparate outcomes between models emphasize the context dependency of their signaling effects and the incomplete picture of their mechanisms. The crosstalk of multiple pathways implicates a complex signaling network, but systems-level integration and population validation are lacking. This restriction also challenges the biological certainty needed to consider these molecules as specific targets for precision intervention.

## 5 Therapeutic strategies targeting ncRNAs

From mechanisms of action to translation, research on the therapeutic use of ncRNAs in CVD has advanced significantly. MiRNA, lncRNA, and circRNA serve as independent but interrelated candidate therapeutic targets that regulate various key pathological processes, such as energy metabolism, inflammation, apoptosis, fibrosis, angiogenesis, and vascular remodeling (Abubakar et al., 2024). Therefore, this chapter outlines therapeutic approaches for each major ncRNA class and how these might be employed to modulate CVD.

### 5.1 Therapeutic potential of miRNA-targeted interventions

The therapeutic utility of miRNA-based drugs is based on their targeting specificity, defined therapeutic roles, and rapid modulation of intracellular homeostasis. These features support the rational design of small RNA-based therapeutics. Emerging evidence from various disease models establishes a central miRNA cohort that modulates cardioprotection, anti-apoptotic signaling, ferroptosis resistance, and angiogenesis in ischemic and remodeling-related cardiovascular injury (Wei Y-L. et al., 2025). P-MSN/miR-199a-5p nanoparticles improve contractile function and inhibit apoptosis during MI and reperfusion injury, indicating potential therapeutic application for long-term post-infarct repair (Chen Y. et al., 2024). A number of natural compounds also exert their protective effects on the heart by miRNA-regulated ferroptosis mechanisms. Ginsenoside Re inhibits I/R-induced ferroptosis through the miR-144–3p/SLC7A11 axis (Ye et al., 2023), and EGCG exerts an anti-ferroptotic effect via miR-450b-5p/ACSL4 pathway signaling (Yu Q. et al., 2023). Altogether, these results suggest combined therapies using drugs formulated with miRNAs and ncRNA-based treatments.

In energy metabolism and mitochondrial function, miR-210 regulates mitochondrial bioenergetics and reactive oxygen species (ROS) flow by targeting glycerol-3-phosphate dehydrogenase, which enhances cardiac performance in I/R models (Song et al., 2022). sEV-miR-486–5p induces angiogenesis through the MMP19/VEGFA cleavage axis. Additionally, engineered exosomal transport has been shown to be both safe and efficacious in non-human primate models of MI (Li Q. et al., 2021). These findings underscore the dual role that miRNAs play in cardiac repair and vascular regeneration. In vascular disease, miR-135b-5p is involved in the pathophysiology of arteriovenous malformations and constitutes a potential diagnostic or therapeutic target (Lee et al., 2024). The application of miR-153–3p increases functional recovery after MI; nHA-CLPs form a stable platform for performing such an application (Zhou G. et al., 2022). In the regulation of cardiac hypertrophy, miR-421 negatively regulates ER stress by repressing FGF13 and preventing disease development (Zhi et al., 2024). Similarly, miR-520c-3p inhibits RelA/p65 and suppresses PDGF-BB-stimulated smooth muscle cell proliferation and migration (Wang et al., 2021). Furthermore, AGPDAR-146a-NPs could deliver miR-146a to restrain oxidative stress and inflammation in atherosclerotic lesions. This method provides a platform for miRNA-based systemic therapies (Li X. et al., 2025).

The focus of miRNA therapeutics is shifting from a one-molecule, one-effect model toward multitiered regulatory and multicascade network models. Their functions extend beyond individual pathways, highlighting their role as integrative mediators at the crossroads of inflammatory, metabolic, and structural remodeling networks. This establishes the foundation for nucleic acid-based therapeutic frameworks tailored to specific pathophysiological subtypes.

### 5.2 Innovative approaches for lncRNA-based interventions

LncRNAs are key players in chromatin modulation, transcriptional control, and regulatory RNA/protein complexes, thereby offering options for intervention upstream of gene regulation in CVD. Therapeutic protocols in progress are primarily concerned with the modification of fibrosis, reduction of inflammatory damage, or normalization of metabolic disturbances and programmed cell death. In fibrosis regulation, lncRNA TARID-loaded lipid nanoparticles upregulate Tcf21 to suppress pathologic cardiac remodeling. This provides a unique RNA-mediated intervention for myocardial fibrosis and demonstrates the feasibility of using extracellular vesicles (EVs) as an effective therapeutic vessel (Zhu D. et al., 2023). Another study identified lncRNA93358 as post-transcriptionally mediated by ZC3H13-associated m6A modification in AMI, acting protectively against inflammation, oxidative stress, and ferroptosis (Cai et al., 2025). These results indicate that the lncRNA- and epigenetics-modulating axis is a potentially druggable regulatory node.

In cardiac hypertrophy and HF models, lncBBR suppresses angiotensin II-induced hypertrophy by regulating the mTOR/AMPK and LC3-dependent autophagy pathway (Zeng et al., 2019). Suppressing lncGASL1 degraded the protective effects of Valsartan on HF phenotypes via PI3K/AKT regulation, indicating renin-angiotensin-aldosterone system (RAAS) inhibitors exert their biological functions through complex, lncRNA-dependent regulatory networks (Zhou J. et al., 2022). Examples of such pathological axes include linc-RMRP, TINCR, and NOS2P3, which are part of independent pathways but converge functionally through competing endogenous RNA (ceRNA) activity mediated by miRNA binding to modulate hypertrophy, inflammation, or apoptosis. Together, these interactions constitute an important lncRNA-miRNA-mRNA regulatory network underlying various cardiovascular phenotypes (Chen J. et al., 2022; Tu et al., 2022; Chen C. et al., 2020).

In AS, lncARF regulates the PI3K/Akt and MAPK pathways and exerts a protective role in resisting plaque formation (Ding et al., 2025). LncCASC11 may inhibit disease progression by downregulating IL-9 and regulating smooth muscle cell proliferation (Tao et al., 2019), whereas RNCR3 can promote inflammatory reactions through endothelial cyclin expression (Hong et al., 2021). These results reveal the complicated system of lncRNA crosstalk in AS with diverse layers, which is dependent on particular cell types. AK006774 is also a critical node for ischemia/reperfusion (I/R) pathology and regulates cell death via the miR-448/Bcl-2 axis in IRI and cardiomyocyte apoptosis (Nie et al., 2021). Kcnq1ot1 promotes AMI injury by restraining miR-466k/miR-466i-5p and facilitating Tead1 (Liao et al., 2020). The finding of H19-mediated regulation of MSC-ATV-Exo-dependent angiogenesis highlights the potential for incorporating lncRNAs into cellular regenerative therapies (Huang et al., 2020b). Altogether, lncRNA-directed methods represent a multifaceted regulatory process ranging from structural interaction to modulatory functions and epigenetic control. These molecules are also important elements in building multitarget therapeutic platforms that could advance the development of individualized treatments. Figure 7 illustrates the manner by which ncRNAs interact with various delivery platforms and pathological targets, underlining the translational model for precision cardiovascular intervention.

**FIGURE 7:**
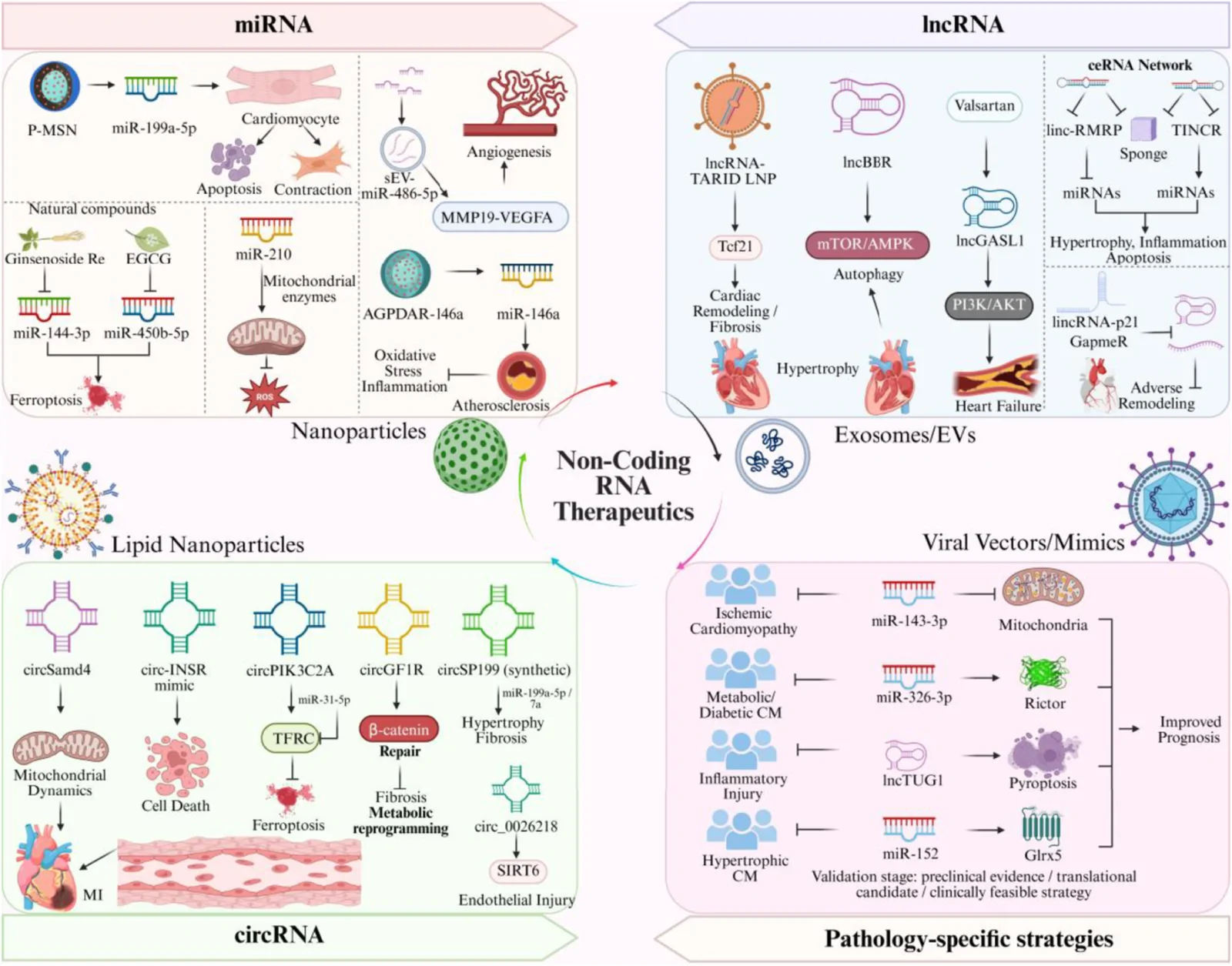
Therapeutic roles of ncRNAs and emerging delivery strategies. This figure integrates the functional targets of ncRNAs with the expanding delivery platforms used in cardiovascular therapy. MiRNAs modulate apoptosis, contractile regulation, angiogenesis, and oxidative stress. This provides therapeutic targets for attenuating tissue injury and metabolic imbalance. Their stability and bioavailability can be enhanced by natural compounds and nanomaterials, which supports therapeutic activity across anti-inflammatory, anti-fibrotic, and ferroptosis inhibiting pathways. CircRNAs modulate mitochondrial function, cell-fate transitions, and metabolic programming, contributing to myocardial repair and vascular remodeling. LncRNAs regulate mTOR/AMPK and PI3K/AKT signaling, as well as the ceRNA network structure, thereby driving hypertrophy, fibrosis, and maladaptive structural remodeling. Exosomes function as natural delivery systems that support cell-specific targeting. Viral vectors and synthetic RNA analogues facilitate efficient intervention in defined pathological states. The validation-stage panel distinguishes preclinical evidence, translational candidates, and clinically feasible strategies, thereby avoiding direct equivalence between experimental efficacy and established clinical applicability. Together, the framework outlines a multidimensional therapeutic scope mediated by ncRNAs and demonstrates their translational potential in precision cardiovascular medicine.

Studies of lncRNA-based therapy are growing quickly but suffer from conceptual divergence. While many studies show effects in fibrosis, inflammation, and vascular remodeling, the majority of mechanistic insights point to single pathways and lack systems-level integration. Divergent responses across experimental models emphasize epigenetic regulation as being highly context-dependent. The development of delivery systems has further expanded therapeutic options, but concerns remain about specificity and safety. In summary, lncRNAs are an important field of precision cardiovascular therapeutics, but actual clinical translation relies on striking a balance between mechanistic consistency and pharmacologic control (Quinones et al., 2025).

### 5.3 Prospects for using circRNAs as therapeutic targets

At present, circRNAs are known for their structural stability, high tissue specificity, and function as miRNA sponges or protein scaffolds, making them appreciated as potential therapeutic targets. Increasing data highlights circRNAs as major regulatory hubs in metabolic regulation, inflammatory signaling, fibrosis, regenerative repair, and cell death pathways. In ischemic injury, circSamd4 protects mitochondrial dynamics by facilitating Vcp translocation and reducing mPTP opening, suggesting it as a potential target for the alleviation of HF after MI (Zheng et al., 2022). CircDhx32 regulates the AdipoR1/AMPK/NF-κB axis and may be a therapeutic target for reducing the inflammatory aspects of ischemia and reperfusion injury (Si et al., 2025). DOX-induced cardiomyocyte death can be ameliorated by overexpression of Circ-INSR, which protects heart function; this also indicates that combination therapy of circRNA mimetics with viral delivery platforms is possible in therapeutic formulations (Lu et al., 2022).

In ferroptosis-mediated AMI, circPIK3C2A induces iron-dependent cell death by miR-31–5p sequestration and TFRC upregulation; this may represent a molecular axis with therapeutic implications in cardiac iron overload diseases (Miao et al., 2024). CircMIRIAF promotes MI/RI injury via sponging miR-544 to raise WDR12. This finding highlights a circRNA node that can be targeted in reperfusion therapy (Yin et al., 2024). In the physiological and pathological processes of cardiac regeneration and remodeling, circIGF1R promotes recovery after MI via regulating the DDX5/β-catenin pathway (Shan et al., 2024). This molecule reprograms cellular metabolism by inhibiting glycolysis, which remarkably decreases fibrotic load, identifying a new therapeutic target for pathological remodeling (Schmidt et al., 2025). Circ_0002295 promotes myocardial fibrosis through regulation of the miR-1287/CXCR2 axis, serving as a target for anti-fibrotic treatments (Ma GB. et al., 2023). In the context of atherogenesis and vascular inflammation, circRNAs are emerging as players in more complex regulatory circuits. Downregulation of circRNA-LONP2 by LSS inhibits endothelial inflammation via the miR-200a-3p/Nrf2/HO-1 axis (Wang R. et al., 2024). Circ_0026218 increases SIRT6 levels and alleviates ox-LDL-induced endothelial injury (Yang L. et al., 2023). CircHIF1α is involved in the epigenetic regulation of AS by mediating the miR-199a-5p/SIRT1 axis, thus providing new targets for anti-inflammatory and antioxidative treatments (Qiao et al., 2023).

In nerve-cardiovascular crosstalk, circ_HECTD1 acts as a sponge for miR-27a-3p to reduce oxygen-glucose deprivation/reoxygenation (OGD/R) injury in stroke via FSTL1. This discovery implies that circRNA-targeted treatments can potentially be used for comorbidities affecting the heart and the brain (Zhang Z. et al., 2021). With their roles being further delineated across various pathological processes, circRNAs are emerging as well-conserved structural molecules endowed with complex regulatory functions. Their modes of action, as miRNA sponges, scaffolds for protein assembly, and platforms for metabolic control, identify them as a complement to other classes of ncRNAs. As a result, these molecules serve as the hubs of multidimensional nucleic acid structures for therapeutic platforms.

The study of ncRNAs as potential therapeutic targets is advancing from mechanism discovery to translational implementation. Throughout myocardial ischemia, HF, AS, and pathways orchestrating structural remodeling, various subfamilies of ncRNAs serve as defined regulatory nodes and can be amenable to inhibition, replacement, or structural mimesis. Preclinical safety and efficacy have been demonstrated in rodent as well as large animal models. In addition, several miRNA-targeted agents have advanced to early-phase clinical trials where they have shown acceptable short-term safety and preliminary biological or functional signals (Shah and Giacca, 2022). Table 6 summarizes major therapeutic strategies, molecular targets, and associated clinical evidence. Thus, if there are translational implications, they should be interpreted with caution, as most *in vitro* and animal studies only support preclinical feasibility, while human trials or patient-based studies provide more definitive evidence for clinical translation. This review offers a framework to understand RNA-based interventions and the structure by which future translation of precise, patient-tailored therapy will be realized.

**TABLE 6:** NcRNA therapeutic and clinical translation research progress.

Therapeutic target	Delivery system/Therapeutic method	Model (Animal or Clinical)	Major therapeutic effects	Translational potential	References
lncRNA CARDINAL	Gene knockout, overexpression	Mouse (animal model)	Inhibits cardiac hypertrophy and abnormal protein translation	Potential therapeutic for cardiac hypertrophy and HF	(He et al., 2024)
miR-199a-5p (targets AGTR1, MARK4)	P-MSN nanoparticles + peptides, intravenous/myocardial injection	Male rat (animal model)	Reduces myocardial injury, protects cardiac function, anti-apoptotic	Long-term MI repair therapeutic potential	(Chen et al., 2024a)
miR-222	TeEV + GelMA hydrogel, injectable patch	Mouse IRI model + neonatal rat cardiomyocytes (NRCMs) (in vitro model)	Alleviates acute IRI, improves post-surgical cardiac remodeling	New strategy potential for myocardial IRI treatment	(Wang et al., 2025c)
CircITCH	Adeno-associated virus serotype 9 (AAV9) overexpression	Mouse model + human induced pluripotent stem cell-derived cardiomyocytes (hiPSC-CMs) (in vitro experiment)	Reduces cardiomyocyte injury and dysfunction	Therapeutic potential	(Han et al., 2020)
miR-450b-5p/ACSL4 axis	EGCG intervention + miR-450b-5p overexpression or knockdown	In vitro hypoxic cardiac model, AMI mouse model	Reduces ferroptosis, improves myocardial ischemia injury	Cardiovascular therapeutic value	(Yu et al., 2023a)
miR-185–5p	Exosome-derived liposome delivery inhibitor	DOX-induced DCM mouse and cell model	Improves cardiac function, reduces apoptosis and copper death	Targeted delivery for HF therapy	(Xu et al., 2025c)
miR-122–5p/BCL2 axis	Exosome-mediated transport	Clinical transcatheter aortic valve replacement (TAVR) patients, mouse model	Modulates cardiomyocyte apoptosis and LVEF	Cardiac function modulation potential	(Hosen et al., 2022)
miR-34a/Wnt5a pathway	Evodia rutaecarpa drug intervention	High-fat diet-induced rat AS model	Improves inflammation, oxidative stress, and blood lipids	Potential for anti-AS therapy	(El Zouka et al., 2024)
SNHG17/miR-34a/SIDT2 pathway	Oral DHM small molecule intervention	Diabetic mouse and HL-1 cell model	Improves heart function, reduces inflammation and fibrosis	Candidate drug for DCM	(Xiao et al., 2024)
lncRNA AK156373 and miR-204–5p/CXCR2	Targeted myocardial necrosis small interfering RNA (siRNA) nanoparticles	MI mouse model and hypoxic cardiomyocytes	Reduces infarction area, improves cardiac function	Preclinical strategy for siRNA application	(Gao et al., 2025)
lncRNA HCG11/miR-224–3p/JAK1	HCG11 knockdown/miR-224–3p overexpression	ox-LDL-induced HUVEC cell model	Inhibits pyroptosis and inflammation, restores vitality	Diagnostic or therapeutic target for AS	(Zhou and Song, 2023)
linc-RMRP/miR-1	linc-RMRP knockdown or miR-1 intervention	PE-induced cardiac hypertrophy model	Suppresses cardiac hypertrophy response	Therapeutic potential for cardiac hypertrophy	(Chen et al., 2022a)
miR-495–3p/Pum2	miR-495–3p silencing intervention	Aortic constriction rats and Ang II cell model	Alleviates hypertrophic response	Therapeutic potential for hypertrophy	(Yu et al., 2024b)
miR-19a-3p/miR-19b-3p	Dietary weight-loss intervention	Clinical population (POUNDS Lost study)	miR-19 decrease associated with reduced atherosclerotic cardiovascular disease (ASCVD) risk	Potential for CVD risk prediction and intervention	(Xue et al., 2024)
lncRNA HOTAIR/SIRT3	Overexpression (HOTAIR or SIRT3)	AC16 cardiomyocytes H/R model	Improves cell vitality, suppresses apoptosis	Potential myocardial I/R protection	(Liu et al., 2023)
miR-503/Apelin-13	AntagomiR-503 injection	Mouse TAC cardiac fibrosis model	Improves cardiac function, suppresses fibrosis	Potential for HF therapy	(Zhou et al., 2016)
miR-483–3p/Cdk9	Lentiviral miR-483–3p overexpression	AMI mouse model	Reduces infarction area, inhibits apoptosis	Potential for myocardial protection	(Xue et al., 2023b)
lncRNA LSINCT5/miR-222	siLSINCT5 transfection	HR-treated AC16 cells, human MI patient plasma	Suppresses apoptosis, enhances cell activity	Potential for MIRI prevention and treatment	(Tong et al., 2021)
lncRNA HOTAIR/miR-17–5p/STAT3	Propofol (PPF) preconditioning	Rat MIRI model, H9c2 cell H/R model	Inhibits apoptosis, reduces myocardial injury	MIRI protection potential	(Chen et al., 2021f)
TY1 inhibition of cGAS/STING pathway	Intravenous or oral micelle formulation	Obese hypertensive HFpEF mouse model	Anti-inflammatory, anti-fibrosis, improves cardiac function	High clinical translation potential	(Miyamoto et al., 2025)
HOTAIR/miR-17–5p/STAT3	PPF preconditioning	AMI rat model	Improves cardiac function, reduces infarction area	Clinical application potential	(Li et al., 2020b)
HOTAIR/miR-519d-3p	HOTAIR overexpression or miR-519d-3p downregulation	MI rat model, hypoxic cardiomyocytes	Reduces cardiomyocyte apoptosis	Potential therapeutic target	(Zhang et al., 2019)
lncRNA ZFAS1/miR-138–5p/SESN2	ZFAS1/SESN2 overexpression vector intervention	Septic myocardial injury rats and H9C2 cells	Reduces myocardial injury and inflammation	Potential therapeutic value	(An et al., 2021)
TUG1/miR-30b-3p/Brd4	TUG1 knockdown or miR-30b-3p overexpression	ApoE−/− AS mouse model	Reduces inflammation and cardiomyocyte apoptosis	Potential therapeutic value	(Li et al., 2023d)
Sirt1-AS/Sirt1	Sirt1-AS overexpression or knockdown intervention	TAC-induced cardiac hypertrophy mouse model	Improves myocardial hypertrophy	Potential therapeutic value	(Wei et al., 2025b)
H19/miR-675/PA2G4	H19 knockdown intervention	DOX-induced DCM rats	Improves ventricular structure and function	Potential therapeutic value	(Zhang et al., 2017c)
HB-EGF/siRNA	Antibody-mediated siRNA delivery system	Hbegf hz/hz; Apoe−/− mouse model	Selective gene silencing	Potential clinical application value	(Tsuchida et al., 2018)
miR-210	MSC-derived exosome myocardial injection	Coronary ligation mouse MI model, hypoxic cardiomyocyte model	Reduces infarction area, improves cardiac function	Myocardial protection and translation potential	(Cheng et al., 2020)
miR-208a/miR-499	Antagomir-208a, antagomir-499 treatment	Mechanical stretch human cardiac fibroblasts; rat volume overload model	Suppresses apoptosis, modulates Bcl-2 expression	Cardiac protection potential	(Chua et al., 2021)
miR-136–5p	LED-Red light stimulation	MI mouse model, cultured cardiomyocytes	Promotes myocardial regeneration, reduces fibrosis	Therapeutic potential for ischemic cardiomyopathy	(Gao et al., 2022)

NcRNA-based therapeutics are moving from the laboratory into a translational context, although establishing mechanisms for such molecules remains an outstanding challenge. Interventions targeting miRNAs affect diverse signaling cascades; however, their efficacy is constrained by delivery effectiveness and unintended secondary effects. LncRNAs and circRNAs offer structural and regulatory advantages; however, their therapeutic use remains at an early stage and requires better characterization of delivery, dosing, pharmacokinetics, and immunogenicity. While broader applicability to different pathological processes makes the modulation of several pathways beneficial, it also creates a more complex understanding and less clear clinical effect in terms of safety. Future development will depend less on expanding RNA target lists than on the prediction and control of intervention strategies that may help redefine cardiovascular therapy (Nappi, 2024).

## 6 ncRNAs and the individualization of cardiovascular therapy

NcRNAs exhibit context-dependent expression profiles in CVDs. These profiles integrate disease subphenotypes and interindividual variation. These characteristics position ncRNAs as pivotal players in precision therapy models (Zhang W. et al., 2025). In this chapter, we provide an overview of the rationale for including ncRNAs in personalized cardiology, detailing precision applications, the mechanisms by which variability arises on a patient-by-patient basis, and future therapeutic perspectives.

### 6.1 Applications of ncRNAs in precision medicine

The application of ncRNAs in cardiovascular therapy is evolving from early single-molecule interventions to strategies tailored based on differential pathological subtypes. Their range of regulation, including energy metabolism, mitochondrial homeostasis, myocardial hypertrophy, inflammatory signaling, and programmed cell death, offers molecular entry points that are congruent with patient-specific disease mechanisms. In the setting of maladaptive remodeling and hypertrophy, HHQ16, a derivative of Astragaloside IV, neutralizes pathologic hypertrophy and infarction-induced HF by inducing the degradation of lncRNA4012/9,456. This illustrates that the interaction interfaces of small molecules and lncRNAs can be utilized for precision targeting (Wan et al., 2023). The LncKCND1/YBX1 axis is an important regulator of mitochondrial performance and a hypertrophic signal inhibitor; depletion of YBX1 nullifies the protection by LncKCND1, demonstrating that YBX1 serves as a downstream node exclusively involved in certain pathological conditions (Yang R. et al., 2023).

Briefly, in IRI, miR-143–3p regulates the Bcl-2-mediated mitochondrial pathway; inhibition of miR-143–3p attenuates early apoptotic loss and appears as an option for early intervention in ischemic cardiomyopathy (Lu et al., 2023). The lincRNA-p21–targeted GapmeR antisense oligonucleotide inhibits cardiac hypertrophy and adverse remodeling, providing additional evidence that the targeting of lncRNAs can be a tangible strategy in structural heart disease (Wang Y. et al., 2024). The synthetic circSP199a specifically sponges miR-199a-5p/-3p to increase metabolic regulators (including PGC-1α, Rb1, SIRT1, and Smad1) and anti-fibrotic pathway genes, followed by the alleviation of hypertrophy and fibrosis as a typical example of precision modulation mediated by circRNA (Wu et al., 2025). In pressure overload models, the miR-152/Glrx5 axis protects cardiac function, rendering a mechanistic framework for molecular subtyping strategies in the management of HF (Larocca et al., 2020).

In diseases with an altered calcium cycle, downregulation of lncRNA Gm17501 diminishes the expression levels of key calcium-cycling proteins, suggesting a cytoplasmic regulatory function in maintaining contractile performance (Liang et al., 2022). LncTUG1, via the miR-186–5p/XIAP axis, suppresses NLRP3 inflammasome-mediated pyroptosis and thus attenuates injury (Zhou et al., 2021a). The specificity of ncRNA actions is also highlighted in metabolic phenotypes. Senescent adipose tissue‐derived exosomes amplify diabetic metabolic dysfunction by transferring miRNA‐326–3p to the myocardium, which represses Rictor. Reversal of this pathologic progression is observed when adipose tissue senescence is decreased (Lin et al., 2022). Exosomes secreted by dendritic cells transport miR-203–3p, which inhibits macrophage cathepsin S and represses the development of AS. This represents patient-specific immune-mediated modulation (Lin et al., 2021). Besides, the suppression of miR-181a-5p also inhibits oxidative stress and inflammation through targeting XIAP (Zhou et al., 2021b). Together, these studies provide evidence that separate ncRNAs can characterize molecular subtypes related to distinct disease contexts and interindividual heterogeneity. Thus, these findings give a mechanistic explanation for precision cardiovascular medicine.

Research on ncRNAs in cardiovascular precision medicine is advancing from mechanistic study to interventions corresponding to different subtypes (Caporali et al., 2024). However, these effects differ greatly from one pathological context to another. The same molecule may exert opposing functions in ischemia versus hypertrophic models; this underscores that the function is determined by the tissue context and by the type of stress. While their capacity to control more than one target at a time mediates systems-level regulation, it also raises questions regarding off-target perturbations and the dose-response relationship. Delivery efficiency, spatial and temporal specificity, immunogenic responses, safety, and clinical validation remain principal obstacles toward translation (Nappi, 2024; Abdul-Rahman et al., 2024). In summary, ncRNAs open up a novel approach to precision therapy; moving them into clinical practice will require convergence between mechanistic understanding and technological advances.

### 6.2 Factors shaping individual responses

Interindividual variability restricts the clinical utility of ncRNAs, which is affected not just by genomic background but also by disease status, tissue type, metabolic condition, environmental exposure, and population-level characteristics (Zhang J. et al., 2026). CHF has shown positive correlations among circulating miRNAs and changes in clinical status during the first 48 h post-admission, suggesting that time-dependent, prognostic markers based on circulating miRNA levels are related to patient-specific features (Vegter et al., 2016). MiRNA reaction is also affected by lifestyle. In exercise-based interventions, aerobic training during pregnancy raises plasma levels of miR-21–3p and prevents the decrease in explosive strength, indicating that behavior can modulate miRNA profiles (Candia et al., 1985). In AS, decreased miR-483–5p is associated with the morphological regression of lesions and may serve as a sensitive indicator of response to therapy (Rehberger Likozar et al., 2025). In HFpEF, circulating miR-181c can discriminate responders from non-responders to exercise training, which can be used as molecular evidence for individual rehabilitation (Gevaert et al., 2021). NcRNAs are also temporally and spatially specific: many miRNAs rise acutely within tissues, while plasma levels increase primarily during the subacute or chronic phases. Moreover, hypoxia and reoxygenation promote cardiomyocytes to secrete specific miRNAs through exosomal release (Danielson et al., 2018). This temporal, tissue-context, and environmental-responsive dynamic regulation provides a mechanism underlying the wide inter-individual variability in therapeutic responses.

In terms of pharmacological response, platelet miR-107 enhances clopidogrel resistance via P2Y12 regulation, and this result further confirms the potential role of miRNAs as clinical predictors for antiplatelet therapy (Zhang Q. et al., 2022). Regulatory networks are also modified by environmental exposures. The short-term cytokine effects of PM2.5 may be mediated through the modulation of specific miRNAs (Chen R. et al., 2018). Patients with acute HF who develop worsening renal function have significantly lower circulating miRNA levels, and miR-199a-3p is the most powerful predictor (Bruno et al., 2016). Metabolic status also affects miRNA profiles. Adipose tissue-derived miRNAs exhibit depot-specific expression patterns in obese individuals receiving liraglutide that are associated with intraoperative glycemia. This indicates that miRNA regulation has a metabolic aspect (Iacobellis et al., 2025). Regarding circulating miRNAs and mortality risk in NSTE-ACS, circulating miR-3135 b and miR-28–3p are candidate markers for further evaluation (Wang et al., 2017). For risk prediction of HF rehospitalization, circulating miR-132 improves model discrimination and suggests novel approaches for clinical stratification (Masson et al., 2018). Taken together, these findings suggest that ncRNAs have value as both diagnostic and therapeutic targets because they are sensitive to personal variability. This structure provides a framework for a model which incorporates patient-specific expression, disease stage response, and heterogeneity in treatment effects.

The role of ncRNAs is highly heterogeneous among individuals; expression levels and effects on target genes are dependent upon genetic background, metabolic status, disease severity, and environmental habits. The same molecule may also serve divergent or even opposite biological functions under physiological or therapeutic circumstances, thereby becoming less suitable as a universal biomarker. Although the spatial and temporal range of miRNAs suggests that they hold promise for precision medicine, it can hamper interpretation. Insufficient clinical metadata and a lack of standardized analytical platforms still remain obstacles to their predictive biomarker applications (Lakkisto et al., 2023). As a result, there is an urgent need for a systematically integrated and longitudinally validated individual response atlas of ncRNAs to further explore their clinical value.

### 6.3 Directions for individualized RNA-based intervention

Personalized ncRNA-based intervention is now evolving from a linear model of biomarker, mechanism, and therapy into a dynamic feedback system focused on three parallel strategies: functional promotion, risk stratification, and target inhibition. MiR-21 acts downstream of Per2 and is upregulated under light-induced cardioprotection (Bartman et al., 2017). Distinctly, circulating miR-124–3p predicts neurological outcomes after cardiac arrest (Devaux et al., 2016). These reports reveal the interaction between ncRNA signals, circadian regulation, and stress physiology as adaptable regulatory processes. Temporal patterns of multi-signature miRNAs display distinct rates of remodeling after MI. This offers molecular signals for early risk prediction and personalized therapeutic strategies (Eyyupkoca et al., 2022). On the pharmacological side, RNA-targeted lipid-modifying therapies illustrate how molecular susceptibility profiles may be incorporated into individualized cardiovascular risk management, although this concept should be distinguished from ncRNA-guided intervention.

CDR132 L suppresses miR-132 to improve HF phenotypes and lower NT-proBNP levels (Täubel et al., 2021). The HF-REVERT trial was designed to evaluate the efficacy and safety of CDR132 L for preventing or reversing cardiac remodeling in patients with post-MI HF, and its results were expected to inform subsequent outcome trials (Bauersachs et al., 2024). The upregulation of miR-423–5p has been identified as a risk factor in heart failure with mid-range ejection fraction (HFmrEF) and could be useful to monitor synergy between dynamic molecular changes and rehabilitation (Huang Y. et al., 2022). At an interventional level, the concomitant application of agents blocking exosomes and si-HIF-1α shows that the lncTUG1/HIF-1α/VEGF-α pathway can be regulated to achieve cardioprotection post-percutaneous coronary intervention (PCI) (Dang et al., 2023). Additionally, the fact that IL-6 and TNF-α can be regulated by miR-146a offers a measurable marker for stratification of risk in AS progression (Huang et al., 2020a). As a result, ncRNAs are not just molecular modifiers but also play pivotal roles in the knowledge base of cardiovascular precision medicine. The combination of dynamic imaging, epigenetic control, and targeted delivery into one single system might provide a closed-loop strategy that connects mechanistic intervention with personalized therapeutic response. Figure 8 represents how ncRNAs serve as a structural foundation for the coherent integration of molecular heterogeneity at the patient level and for transforming it into specific individualized treatment strategies.

**FIGURE 8:**
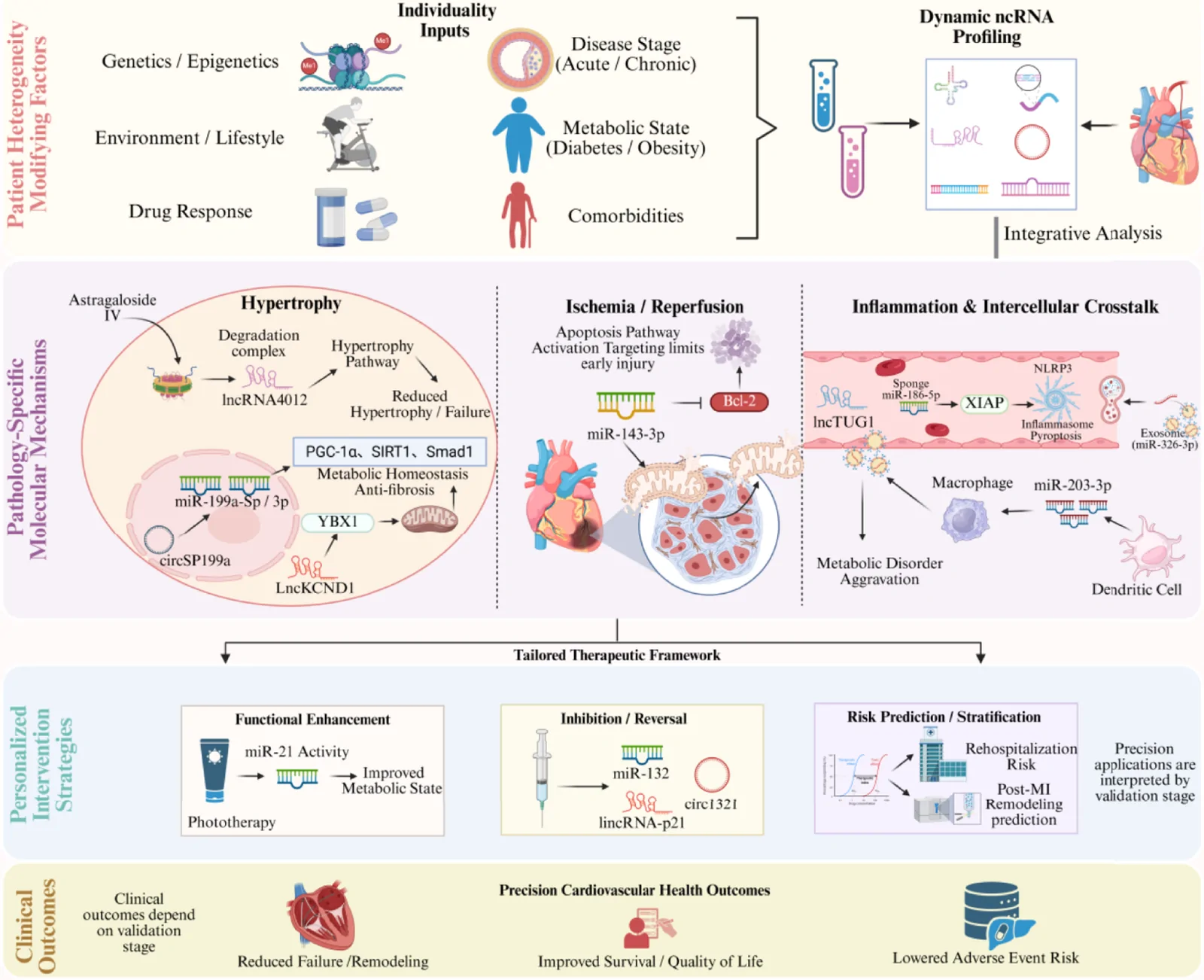
NcRNA-driven frameworks for precision and individualized cardiovascular therapy. This figure outlines a precision-medicine framework centered on ncRNAs, integrating heterogeneity across genetic and epigenetic backgrounds, metabolic states, disease stages, and comorbid conditions into a continuous, dynamic molecular readout. In cardiac hypertrophy, lncRNA4012, circSP199a, and LncKCND1 coordinate metabolic homeostasis and fibrotic responses; in ischaemia–reperfusion injury, miR-143–3p contributes to early programming of mitochondrial and apoptotic signalling; in inflammatory and immune-mediated contexts, lncTUG1 and miR-203–3p regulate NLRP3 activation, metabolic imbalance, and intercellular communication routes. These layered mechanisms provide molecular anchors for individualized intervention strategies spanning metabolic correction, antifibrotic therapy, reversal of structural remodeling, risk prediction, and patient stratification. The clinical-outcome layer emphasizes that these individualized strategies require validation-stage interpretation before they can be considered clinically actionable. Overall, the framework illustrates how ncRNA-based molecular profiles can be translated into actionable, patient-specific therapeutic directions.

NcRNA-targeted therapeutics are pushing cardiovascular precision medicine from static molecular features to a dynamic program of regulation. Instead of focusing on single pathways, ncRNAs integrate regulatory functions across inflammatory, metabolic, and structural remodeling networks. Their diverse signaling profiles and asynchronous temporal patterns, however, render therapeutic responses highly context-dependent (Zhang W. et al., 2025). In some pathological subtypes, single ncRNAs may even show opposite effects, indicating the complexity of the regulatory network. More directed treatments like CDR132 L underline the therapeutic flexibility of this modality. Nevertheless, issues of dosage control, delivery efficiency, functional redundancy, and microenvironmental dependence still prevent clinical translation. NcRNAs are considered molecular switch controllers connecting circadian physiology, environmental stressors, and cell-fate programs. Their multi-omic signatures and adaptive responses are shaping the paradigm of cardiovascular therapeutics: from static stratification toward a dynamic and predictive framework (Zhang W. et al., 2025).

## 7 Discussion

Studies on ncRNAs are reshaping the way we think about cardiovascular biology. The cumulative regulatory network composed of miRNAs, lncRNAs and circRNAs intertwines metabolic stress, inflammation, cell death and fibrosis in the course of cardiovascular disorders from local insults to systemic disturbance (Liao et al., 2023). However, evidence is still lacking in many areas. The majority of works only consider single types of cells or decoupled pathway nodes, thus capturing little about the intricate dynamics in such processes as intercellular communication, spatiotemporal coordination and tissue-specific context. The observation that an ncRNA can elicit divergent responses at different disease stages implies the non-linear nature of its regulatory role, suggesting regulation by environmental stimuli, metabolic status, and stress signaling within a dynamic system. An additional obstacle is intersubject variability: genetic background, drug exposure, inflammatory load, lifestyle, and cohort-related factors all influence ncRNA expression profiles (Zhang W. et al., 2025; Searles, 2024). Lack of a standardization of multicentre analysing platforms hampers some comparison between datasets and has an unfavorable effect on the biological certainty of statistical associations. Delivery is the limiting factor in many technologies, and this inhibits translation. Mechanistically, while nanoparticles and exosome-based systems can function efficiently in animal studies, it is not well understood how they interact with the immune system and are distributed within tissues or their long-term safety. Early-stage evidence from miRNA inhibitors including CDR132 L indicates therapeutic potential, although it does not define the complete spectrum of clinical feasibility (Täubel et al., 2021).

The advancement of multi-omics has transformed the way investigations are framed. Single-cell sequencing and spatial transcriptomics make it possible to reconstruct the spatiotemporal organization of ncRNA regulation throughout the disease continuum. This provides insights into nodal points of signal convergence and network-level drivers. These innovations provide a step forward, reinforcing the transition from descriptive association to mechanistic models. MiRNA mimics, antisense oligonucleotides, lncRNA-targeting regulators, and circRNA analogues form therapeutic platforms for nucleic acid-directed intervention (Chen Y. et al., 2024; Wu et al., 2025; Zhou et al., 2021a). The development of delivery systems, such as targeted exosomes (Zhu D. et al., 2023), controlled‐release nanoparticles (Zhou J. et al., 2022), and biocompatible viral vectors, has extended the range of RNA therapeutic structures beyond “traditional” pharmacological entities. Composite approaches integrating RAAS inhibitors, antiplatelet agents, and metabolic modulators are predicted to enter the era of precision medicine. This will move from single-target strategies towards system-level allocation. Concurrently, patterns of ncRNA expression are beginning to delineate the pathologic heterogeneity in HF, ACSs, and AS. Together, these form the platform for RNA-informed reclassification of disease. With the advent of high-sensitivity analytical methods combined with wearable sensor technologies (Zhang S. et al., 2025), ncRNAs are expected to be integrated into real-time risk assessment and dynamic disease-monitoring technology.

The study of ncRNAs is transitioning from the observation of signals to manipulation of regulatory circuitry. This scope no longer remains at the level of molecular modification but covers the scope between diagnosis, mechanistic definition, therapeutic intervention and disease follow-up (Bartman et al., 2017; Devaux et al., 2016). Moreover, miRNAs have been shown to mediate rapid feedback control of acute responses. LncRNAs regulate the balance of chronic remodeling by means of chromatin regulation and transcriptomic reprogramming, whereas circRNAs, with their superior stability and intercellular mobility, help maintain systemic homeostasis. Collectively, they provide evidence for a layered yet integrated regulatory network of these classes of RNA that coordinate metabolic stress, immune activation, and structural remodeling and which serve as molecular intervention points to stage-specific targeting. Their personalized expression profiles highlight genetic/metabolic context-dependent interrelationships, features that also position ncRNAs as molecular intermediates for patient-specific pathogenic drivers and an important step toward precision medicine (Zhang W. et al., 2025). But the field is fraught with contrasting paradigms as it moves from one theory to another. While ncRNAs go a long way toward explaining the systemic molecular logic of CVD, ongoing work remains limited by mechanistic compartmentalization, reliance on models and narrow translational validation. The bidirectional context-dependent effects of miRNAs, lncRNAs, and circRNAs reflect the dynamic scenery of these networks; however, analytical heterogeneity and enduring obstacles in delivery are still hampering the clinical depth. Multi-omics-based and nucleic acid-derived therapeutics will redefine the scope of investigator-initiated studies. Whether ncRNAs can ultimately progress from associative signaling to a fully regulable therapeutic system remains the fundamental question and opportunity of precision cardiovascular medicine (Zhang W. et al., 2025).

The significance of ncRNAs extends far beyond their role as biomarkers. Their complex dynamic expression profiles and multiple pathways interactions comprise an intricate regulatory network, which provides a new insight for precision medicine in the field of cardiovascular pathophysiology. With a more precise understanding of epigenetic regulation, tissue-specific readouts, and integration of multi-omics data, ncRNAs may help connect mechanistic interpretation with personalized therapeutic matching (Baccarelli and Ordovás, 2023). Consequently, these advances increase their capabilities as a feasible tool to redefine disease stratification, risk predictors and treatment strategies.

## Glossary

AAV9: adeno-associated virus serotype 9
ACS: acute coronary syndrome
AF: atrial fibrillation
AMI: acute myocardial infarction
AS: atherosclerosis
ASCVD: atherosclerotic cardiovascular disease
ASPN: asporin
AUC: area under the curve
CAA: coronary artery aneurysm
CAC: coronary artery calcification
CAD: coronary artery disease
CAS: carotid atherosclerosis
CAVD: calcific aortic valve disease
ceRNA: competing endogenous RNA
CHF: chronic heart failure
circRNA(s): circular RNA(s)
CRISPR: clustered regularly interspaced short palindromic repeats
CT: computed tomography
CVD(s): cardiovascular disease(s)
DCM: diabetic cardiomyopathy
DOX: doxorubicin
ECL: electrochemiluminescence
ECM: extracellular matrix
EndoMT: endothelial-to-mesenchymal transition
EV(s): extracellular vesicle(s)
FRET: fluorescence resonance energy transfer
H/R: hypoxia/reoxygenation
HF: heart failure
HFmrEF: heart failure with mid-range ejection fraction
HFpEF: heart failure with preserved ejection fraction
HFrEF: heart failure with reduced ejection fraction
hiPSC-CMs: human induced pluripotent stem cell-derived cardiomyocytes
HTN: hypertension
HUVEC(s): human umbilical vein endothelial cell(s)
I/R: ischemia/reperfusion
IRI: ischemia-reperfusion injury
LAA-stroke: large artery atherosclerosis ischemic stroke
lncRNA(s): long non-coding RNA(s)
LOD: limit of detection
MACE: major adverse cardiovascular events
MI: myocardial infarction
MIRI: myocardial ischemia-reperfusion injury
miRNA(s): microRNA(s)
mPTP: mitochondrial permeability transition pore
mRNA(s): messenger RNA(s)
ncRNA(s): non-coding RNA(s)
NRCMs: neonatal rat cardiomyocytes
NSTE-ACS: non-ST elevation acute coronary syndrome
NSTEMI: non-ST segment elevation myocardial infarction
NT-proBNP: N-terminal pro-B-type natriuretic peptide
NYHA: New York Heart Association
OGD: oxygen-glucose deprivation
OGD/R: oxygen-glucose deprivation/reoxygenation
OSA: obstructive sleep apnea
oxLDL: oxidized LDL
PAH: pulmonary arterial hypertension
PASMCs: pulmonary arterial smooth muscle cells
PCI: percutaneous coronary intervention
PCR: polymerase chain reaction
PH: pulmonary hypertension
PMI: perioperative myocardial injury
PPF: propofol
RAAS: renin-angiotensin-aldosterone system
RBP(s): RNA-binding protein(s)
RISC: RNA-induced silencing complex
ROS: reactive oxygen species
SERS: surface-enhanced Raman scattering
sEV(s): small extracellular vesicle(s)
siRNA: small interfering RNA
STEMI: ST-segment-elevation myocardial infarction
STOCs: spontaneous transient outward currents
TAC: transverse aortic constriction
TAVR: transcatheter aortic valve replacement
VMC: viral myocarditis
VSMC(s): vascular smooth muscle cell(s)
